# Ribosomal RNA (rRNA) sequences from 33 globally distributed mosquito species for improved metagenomics and species identification

**DOI:** 10.7554/eLife.82762

**Published:** 2023-01-23

**Authors:** Cassandra Koh, Lionel Frangeul, Hervé Blanc, Carine Ngoagouni, Sébastien Boyer, Philippe Dussart, Nina Grau, Romain Girod, Jean-Bernard Duchemin, Maria-Carla Saleh

**Affiliations:** 1 Institut Pasteur, Université Paris Cité, CNRS UMR3569, Viruses and RNA Interference Unit, F-75015 Paris France; 2 Institut Pasteur de Bangui, Medical Entomology Laboratory Bangui Central African Republic; 3 Institut Pasteur du Cambodge, Medical and Veterinary Entomology Unit Phnom Penh Cambodia; 4 Institut Pasteur du Cambodge, Virology Unit Phnom Penh Cambodia; 5 Institut Pasteur de Madagascar, Medical Entomology Unit Antananarivo Madagascar; 6 Institut Pasteur de la Guyane, Vectopôle Amazonien Emile Abonnenc Cayenne French Guiana; University of Colorado Boulder United States; University of Colorado Boulder United States

**Keywords:** mosquito, metagenomics, surveillance, ribosomal RNA, molecular taxonomy, RNA-seq, Other

## Abstract

Total RNA sequencing (RNA-seq) is an important tool in the study of mosquitoes and the RNA viruses they vector as it allows assessment of both host and viral RNA in specimens. However, there are two main constraints. First, as with many other species, abundant mosquito ribosomal RNA (rRNA) serves as the predominant template from which sequences are generated, meaning that the desired host and viral templates are sequenced far less. Second, mosquito specimens captured in the field must be correctly identified, in some cases to the sub-species level. Here, we generate mosquito rRNA datasets which will substantially mitigate both of these problems. We describe a strategy to assemble novel rRNA sequences from mosquito specimens and produce an unprecedented dataset of 234 full-length 28S and 18S rRNA sequences of 33 medically important species from countries with known histories of mosquito-borne virus circulation (Cambodia, the Central African Republic, Madagascar, and French Guiana). These sequences will allow both physical and computational removal of rRNA from specimens during RNA-seq protocols. We also assess the utility of rRNA sequences for molecular taxonomy and compare phylogenies constructed using rRNA sequences versus those created using the gold standard for molecular species identification of specimens—the mitochondrial *cytochrome* c *oxidase I* (COI) gene. We find that rRNA- and COI-derived phylogenetic trees are incongruent and that 28S and concatenated 28S+18S rRNA phylogenies reflect evolutionary relationships that are more aligned with contemporary mosquito systematics. This significant expansion to the current rRNA reference library for mosquitoes will improve mosquito RNA-seq metagenomics by permitting the optimization of species-specific rRNA depletion protocols for a broader range of species and streamlining species identification by rRNA sequence and phylogenetics.

## Introduction

Mosquitoes top the list of vectors for arthropod-borne diseases, being implicated in the transmission of many human pathogens responsible for arboviral diseases, malaria, and lymphatic filariasis (WHO, 2017). Mosquito-borne viruses circulate in sylvatic (between wild animals) or urban (between humans) transmission cycles driven by different mosquito species with their own distinct host preferences. Although urban mosquito species are chiefly responsible for amplifying epidemics in dense human populations, sylvatic mosquitoes maintain the transmission of these viruses among forest-dwelling animal reservoir hosts and are involved in spillover events when humans enter their ecological niches (Valentine et al., 2019). Given that mosquito-borne virus emergence is preceded by such spillover events, continuous surveillance and virus discovery in sylvatic mosquitoes is integral to designing effective public health measures to pre-empt or respond to mosquito-borne viral epidemics.

Metagenomics on field specimens is a powerful method in our toolkit to understand mosquito-borne disease ecology through the One Health lens (Webster et al., 2016). With next-generation sequencing becoming more accessible, such studies have provided unprecedented insights into the interfaces among mosquitoes, their environment, and their animal and human hosts. As mosquito-associated viruses are mostly RNA viruses, RNA sequencing (RNA-seq) is especially informative for surveillance and virus discovery. However, working with lesser studied mosquito species poses several problems.

First, metagenomics studies based on RNA-seq are bedevilled by overabundant ribosomal RNAs (rRNAs). These non-coding RNA molecules comprise at least 80% of the total cellular RNA population (Gale and Crampton, 1989). Due to their length and their abundance, they are a sink for precious next-generation sequencing reads, decreasing the sensitivity of pathogen detection unless depleted during library preparation. Yet the most common rRNA depletion protocols require prior knowledge of rRNA sequences of the species of interest as they involve hybridizing antisense oligos to the rRNA molecules prior to removal by ribonucleases (Fauver et al., 2019; Phelps et al., 2021) or by bead capture (Kukutla et al., 2013). Presently, reference sequences for rRNAs are limited to only a handful of species from three genera: *Aedes*, *Culex*, and *Anopheles* (Ruzzante et al., 2019). The lack of reliable rRNA depletion methods could deter mosquito metagenomics studies from expanding their sampling diversity, resulting in a gap in our knowledge of mosquito vector ecology. The inclusion of lesser studied yet medically relevant sylvatic species is therefore imperative.

Second, species identification based on morphology is notoriously complicated for members of certain species subgroups. This is especially the case among *Culex* subgroups. Sister species are often sympatric and show at least some competence for a number of viruses, such as Japanese encephalitis virus, St Louis encephalitic virus, and Usutu virus (Nchoutpouen et al., 2019). Although they share many morphological traits, each of these species have distinct ecologies and host preferences, thus the challenge of correctly identifying vector species can affect epidemiological risk estimation for these diseases (Farajollahi et al., 2011). DNA molecular markers are often employed to a limited degree of success to distinguish between sister species (Batovska et al., 2017; Zittra et al., 2016).

To address the lack of full-length rRNA sequences in public databases, we sought to determine the 28S and 18S rRNA sequences of a diverse set of Old and New World sylvatic mosquito species from four countries representing three continents: Cambodia, the Central African Republic, Madagascar, and French Guiana. These countries, due to their proximity to the equator, contain high mosquito biodiversity (Foley et al., 2007) and have had long histories of mosquito-borne virus circulation (Desdouits et al., 2015; Halstead, 2019; Héraud et al., 2022; Jacobi and Serie, 1972; Ratsitorahina et al., 2008; Saluzzo et al., 2017; Zeller et al., 2016). Increased and continued surveillance of local mosquito species could lead to valuable insights on mosquito virus biogeography. Using a unique score-based read filtration strategy to remove interfering non-mosquito rRNA reads for accurate de novo assembly, we produced a dataset of 234 novel full-length 28S and 18S rRNA sequences from 33 mosquito species, 30 of which have never been recorded before.

We also explored the functionality of 28S and 18S rRNA sequences as molecular markers by comparing their performance to that of the mitochondrial *cytochrome* c *oxidase subunit I* (COI) gene for molecular taxonomic and phylogenetic investigations. The COI gene is the most widely used DNA marker for molecular species identification and forms the basis of the Barcode of Life Data System (BOLD) (Hebert et al., 2003; Ratnasingham and Hebert, 2007). Presently, full-length rRNA sequences are much less represented compared to other molecular markers. However, given the availability of relevant reference sequences, 28S and concatenated 28S+18S rRNA sequences can be the better approach for molecular taxonomy and phylogenetic studies. We hope that our sequence dataset, with its species diversity and eco-geographical breadth, and the assembly strategy we describe would further facilitate the use of rRNA as markers. In addition, this dataset enables the design of species-specific oligos for cost-effective rRNA depletion for a broader range of mosquito species and streamlined molecular species identification during RNA-seq.

## Results

### Poor rRNA depletion using a non-specific depletion method

**Figure 1.**
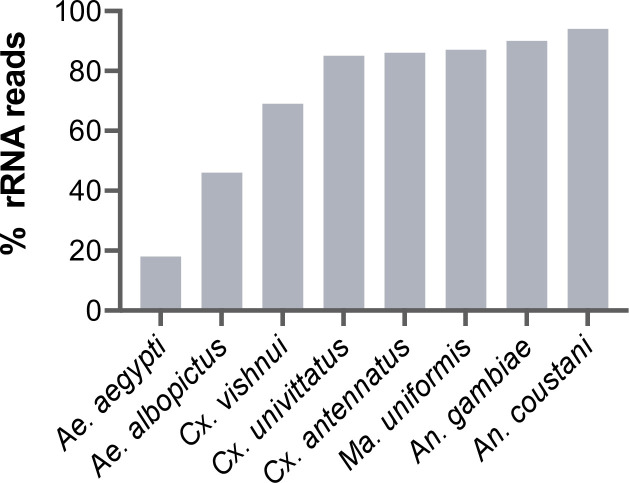
Percentage of rRNA reads in mosquito total RNA sequencing (RNA-seq) data after depletion using probes antisense to *Aedes aegypti* sequences. Pools of five individual mosquitoes from genera *Aedes* (*Ae*), *Culex* (*Cx*), *Mansonia* (*Ma*), and *Anopheles* (*An*) were ribodepleted by probe hybridisation followed by RNase H digestion according to the protocol by Morlan et al., 2012. Y-axis depicts percentages of remaining rRNA reads calculated as the number of rRNA reads over total reads per sample pool. Depletion efficiency decreases with taxonomic distance from *Ae. aegypti* underlining the need for reference sequences for species of interest.

**Figure 2.**
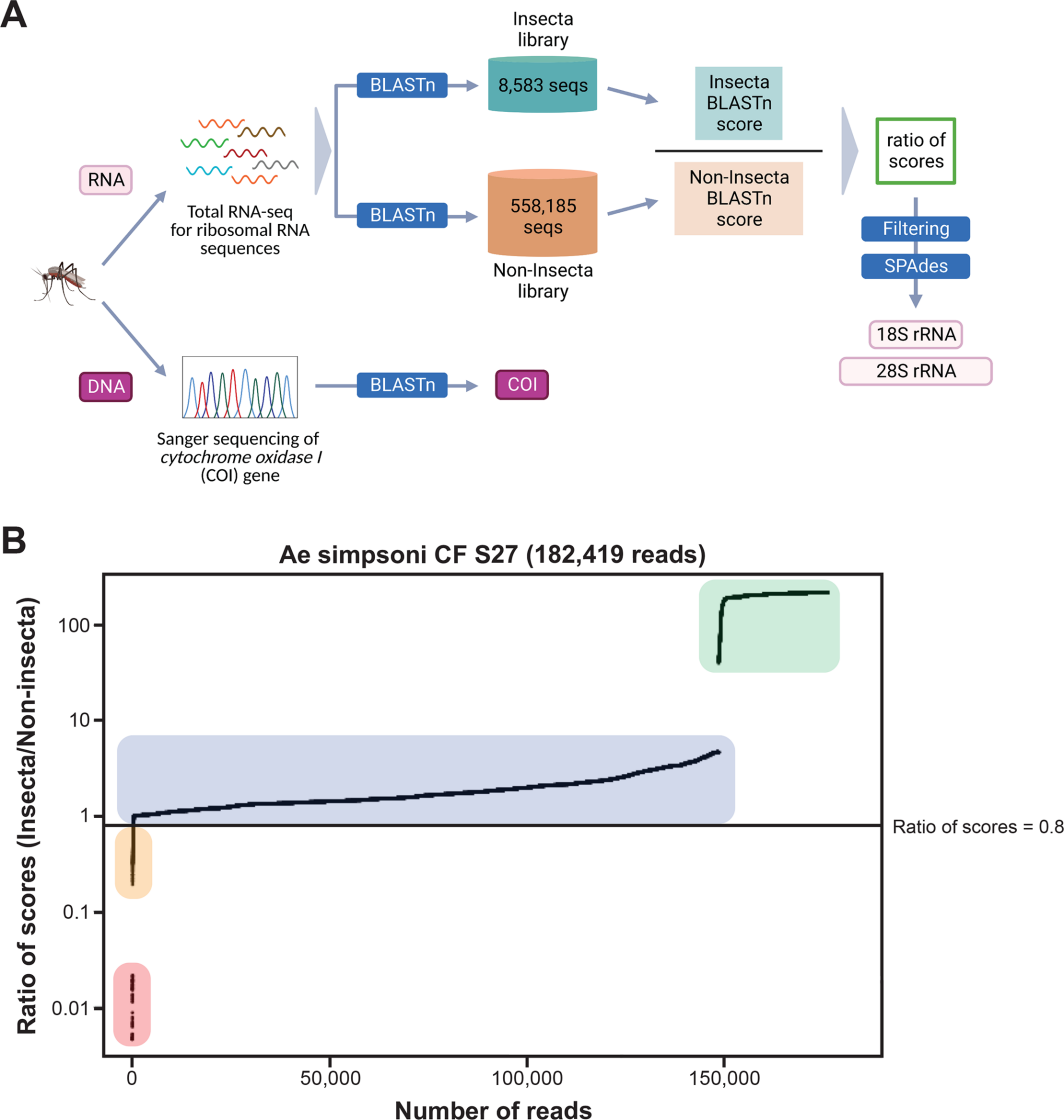
Novel mosquito rRNA sequences were obtained using a unique reads filtering method. (**A**) Schematic of sequencing and bioinformatics analyses performed in this study to obtain full-length 18S and 28S rRNA sequences as well as cytochrome *c* oxidase I (COI) DNA sequences. Nucleic acids were isolated from mosquito specimens for next-generation (for rRNA) or Sanger (for COI) sequencing. Two in-house libraries were created from the SILVA rRNA gene database: Insecta and Non-Insecta, which comprises 8,585 sequences and 558,185 sequences, respectively. Following BLASTn analyses against these two libraries, each RNA-sequencing (RNA-seq) read is assigned a ratio of BLASTn scores to describe their relative nucleotide similarity to insect rRNA sequences. Based on these ratios of scores, RNA-seq reads can then be filtered to remove non-mosquito reads prior to assembly with SPAdes to give full-length 18S and 28S rRNA sequences. Image created with https://biorender.com/. (**B**) Based on their ratio of scores, reads can be segregated into four categories, as shown on this ratio of scores versus number of reads plot for the representative specimen ‘CF S27’: (i) reads with hits only in the Insecta library (shaded in green), (ii) reads with a higher score against the Insecta library (shaded in blue), (iii) reads with a higher score against the Non-Insecta library (shaded in yellow), and (iv) reads with no hits in the Insecta library (shaded in red). We applied a conservative threshold at 0.8, indicated by the black horizontal line, where only reads above this threshold are used in the assembly with SPAdes. For this given specimen, 175,671 reads (96.3% of total reads) passed the ≥0.8 cut-off, 325 reads (0.18% of total reads) had ratios of scores <0.8, while 6,423 reads (3.52%) did not have hits against the Insecta library.

During library preparations of mosquito samples for RNA-seq, routinely used methods for depleting rRNA are commercial kits optimised for human or mice samples (Belda et al., 2019; Bishop-Lilly et al., 2010; Chandler et al., 2015; Kumar et al., 2012; Weedall et al., 2015; Zakrzewski et al., 2018) or through 80–100 base pair antisense probe hybridisation followed by ribonuclease digestion (Fauver et al., 2019; Phelps et al., 2021). In cases where the complete reference rRNA sequence of the target species is not known, oligos would be designed based on the rRNA sequence of the closest related species (25, this study). These methods should deplete reads from the conserved regions of rRNA sequences. However, reads from the variable regions remain at abundances high enough to compromise RNA-seq output. In our hands, we have found that using probes designed for the *Ae. aegypti* rRNA sequence followed by RNase H digestion according to the protocol published by Morlan et al., 2012, produced poor depletion in *Aedes albopictus*, and in Culicine and Anopheline species (Figure 1), in which between 46% and 94% of reads post-depletion were ribosomal. Additionally, the lack of full-length reference rRNA sequences compromises the *in silico* clean-up of remaining rRNA reads from sequencing data, as reads belonging to variable regions would not be removed. To solve this and to enable RNA-seq metagenomics on a broader range of mosquito species, we performed RNA-seq to generate reference rRNA sequences for 33 mosquito species representing 10 genera from Cambodia, the Central African Republic, Madagascar, and French Guiana. Most of these species are associated with vector activity for various pathogens in their respective ecologies (Table 1). In parallel, we sequenced the mitochondrial COI gene to perform molecular species identification of our samples and to comparatively evaluate the use of rRNA as a molecular marker (Figure 2).

**Table 1.** Mosquito species represented in this study and their vector status.

Mosquito taxonomy^‡^	Origin*	Collection site (ecosystem type)	Vector for^†^	Reference
*Aedes (Fredardsius) vittatus*	CF	Rural (village)	ZIKV, CHIKV, YFV	Diallo et al., 2020
*Aedes (Ochlerotatus) scapularis*	GF	Rural (village)	YFV	Vasconcelos et al., 2001
*Aedes (Ochlerotatus) serratus*	GF	Rural (village)	YFV, OROV	Cardoso et al., 2010; Romero-Alvarez and Escobar, 2018
*Aedes (Stegomyia) aegypti*	CF	Urban	DENV, ZIKV, CHIKV, YFV	Kraemer et al., 2019
*Aedes (Stegomyia) albopictus*	CF, KH	Rural (village, nature reserve)	DENV, ZIKV, CHIKV, YFV, JEV	Auerswald et al., 2021; Kraemer et al., 2019
*Aedes (Stegomyia) simpsoni*	CF	Rural (village)	YFV	Mukwaya et al., 2000
*Anopheles (Anopheles) baezai*	KH	Rural (nature reserve)	Unreported	–
*Anopheles (Anopheles) coustani*	MG, CF	Rural (village)	RVFV, malaria	Mwangangi et al., 2013; Nepomichene et al., 2018; Ratovonjato et al., 2011
*Anopheles (Cellia) funestus*	MG, CF	Rural (village)	ONNV, malaria	Lutomiah et al., 2013; Tabue et al., 2017
*Anopheles (Cellia) gambiae*	MG, CF	Rural (village)	ONNV, malaria	Brault et al., 2004
*Anopheles (Cellia) squamosus*	MG	Rural (village)	RVFV, malaria	Ratovonjato et al., 2011; Stevenson et al., 2016
*Coquillettidia (Rhynchotaenia) venezuelensis*	GF	Rural (village)	OROV	Travassos da Rosa et al., 2017
*Culex (Culex) antennatus*	MG	Rural (village)	RVFV	Nepomichene et al., 2018; Ratovonjato et al., 2011
*Culex (Culex) duttoni*	CF	Rural (village)	Unreported	–
*Culex (Culex) neavei*	MG	Rural (village)	USUV	Nikolay et al., 2011
*Culex (Culex) orientalis*	KH	Rural (nature reserve)	JEV	Kim et al., 2015
*Culex (Culex) perexiguus*	MG	Rural (village)	WNV, USUV	Vezenegho et al., 2022
*Culex (Culex) pseudovishnui*	KH	Rural (nature reserve)	JEV	Auerswald et al., 2021
*Culex (Culex) quinquefasciatus*	MG, CF, KH	Rural (village, nature reserve)	ZIKV, JEV, WNV, DENV, SLEV, RVFV, *Wuchereria bancrofti*	Bhattacharya and Basu, 2016; Maquart et al., 2021; Ndiaye et al., 2016; Serra et al., 2016
*Culex (Culex) tritaeniorhynchus*	MG, KH	Rural (village, nature reserve)	JEV, WNV, RVFV	Auerswald et al., 2021; Hayes et al., 1980; Jupp et al., 2002
*Culex (Melanoconion) spissipes*	GF	Rural (village)	VEEV	Weaver et al., 2004
*Culex (Melanoconion) portesi*	GF	Rural (village)	VEEV, TONV	Talaga et al., 2021; Weaver et al., 2004
*Culex (Melanoconion) pedroi*	GF	Rural (village)	EEEV, VEEV, MADV	Talaga et al., 2021; Turell et al., 2008
*Culex (Oculeomyia) bitaeniorhynchus*	MG, KH	Rural (village, nature reserve)	JEV	Auerswald et al., 2021
*Culex (Oculeomyia) poicilipes*	MG	Rural (village)	RVFV	Ndiaye et al., 2016
*Eretmapodites intermedius*	CF	Rural (village)	Unreported	–
*Limatus durhamii*	GF	Rural (village)	ZIKV	Barrio-Nuevo et al., 2020
*Mansonia (Mansonia) titillans*	GF	Rural (village)	VEEV, SLEV	Hoyos-López et al., 2015; Turell, 1999
*Mansonia (Mansonioides) indiana*	KH	Rural (nature reserve)	JEV	Arunachalam et al., 2004
*Mansonia (Mansonioides) uniformis*	MG, CF, KH	Rural (village, nature reserve)	RVFV, *Wuchereria bancrofti*	Lutomiah et al., 2013; Ughasi et al., 2012
*Mimomyia (Etorleptiomyia) mediolineata*	MG	Rural (village)	Unreported	–
*Psorophora (Janthinosoma) ferox*	GF	Rural (village)	ROCV	Mitchell et al., 1986
*Uranotaenia (Uranotaenia) geometrica*	GF	Rural (village)	Unreported	–

* Dengue virus, DENV; Zika virus, ZIKV; chikungunya virus, CHIKV; Yellow Fever virus, YFV; Oropouche virus, OROV; Japanese encephalitis virus, JEV; Rift Valley Fever virus, RVFV; O’Nyong Nyong virus, ONNV; Usutu virus, USUV; West Nile virus, WNV; St Louis encephalitis virus, SLEV; Venezuelan equine encephalitis virus, VEEV; Tonate virus, TONV; Eastern equine encephalitis virus, EEEV; Madariaga virus, MADV; Rocio virus, ROCV.

† Origin countries are listed as their ISO alpha-2 codes: Central African Republic, CF; Cambodia, KH; Madagascar, MG; French Guiana, GF.

‡ Subgenus indicated in brackets.

### rRNA reads filtering and sequence assembly

Assembling Illumina reads to reconstruct rRNA sequences from total mosquito RNA is not a straightforward task. Apart from host rRNA, total RNA samples also contain rRNA from other organisms associated with the host (microbiota, external parasites, or ingested diet). As rRNA sequences share high homology in conserved regions, Illumina reads (150 bp) from non-host rRNA can interfere with the contig assembly of host 28S and 18S rRNA.

Our score-based filtration strategy, described in detail in the Materials and methods section, allowed us to bioinformatically remove interfering rRNA reads and achieve successful de novo assembly of 28S and 18S rRNA sequences for all our specimens. Briefly, for each Illumina read, we computed a ratio of BLAST scores against an Insecta library over scores against a Non-Insecta library (Figure 2A). Based on their ratio of scores, reads could be segregated into four categories (Figure 2B): (i) reads mapping only to the Insecta library, (ii) reads mapping better to the Insecta relative to Non-Insecta library, (iii) reads mapping better to the Non-Insecta relative to the Insecta library, and (iv) reads mapping only to the Non-Insecta library. By applying a conservative threshold at 0.8 to account for the non-exhaustiveness of the SILVA database, we removed reads that likely do not originate from mosquito rRNA. Notably, 15 of our specimens were engorged with vertebrate blood, a rich source of non-mosquito rRNA (Appendix 1—table 1). The successful assembly of complete 28S and 18S rRNA sequences for these specimens demonstrates that this strategy performs as expected even with high amounts of non-host rRNA reads. This is particularly important in studies on field-captured mosquitoes as females are often sampled already having imbibed a blood meal or captured using the human landing catch technique.

We encountered challenges for three specimens morphologically identified as *Mansonia africana* (Specimen ID S33–S35) (Appendix 1—table 1). COI amplification by PCR did not produce any product, hence COI sequencing could not be used to confirm species identity. In addition, the genome assembler SPAdes (Bankevich et al., 2012) was only able to assemble partial length rRNA contigs, despite the high number of reads with high scores against the Insecta library. Among other *Mansonia* specimens, these partial length contigs shared the highest similarity with contigs obtained from sample ‘Ma uniformis CF S51’. We then performed a guided assembly using the 28S and 18S sequences of this specimen as references, which successfully produced full-length contigs. In two of these specimens (Specimen ID S34 and S35), our assembly initially produced two sets of 28S and 18S rRNA sequences, one of which was similar to mosquito rRNA with low coverage and another with 10-fold higher coverage and 95% nucleotide sequence similarity to a water mite of genus *Horreolanus* known to parasitize mosquitoes. Our success in obtaining rRNA sequences for mosquito and water mite shows that our strategy can be applied to metabarcoding studies where the input material comprises multiple insect species, provided that appropriate reference sequences of the target species or of a close relative are available.

Altogether, we were able to assemble 122 28S and 114 18S full-length rRNA sequences for 33 mosquito species representing 10 genera sampled from four countries across three continents. This dataset contains, to our knowledge, the first records for 30 mosquito species and for seven genera: *Coquillettidia*, *Mansonia*, *Limatus*, *Mimomyia*, *Uranotaenia*, *Psorophora,* and *Eretmapodites*. Individual GenBank accession numbers for these sequences and specimen information are listed in Appendix 1—table 1.

### Comparative phylogeny of novel rRNA sequences relative to existing records

**Figure 3.**
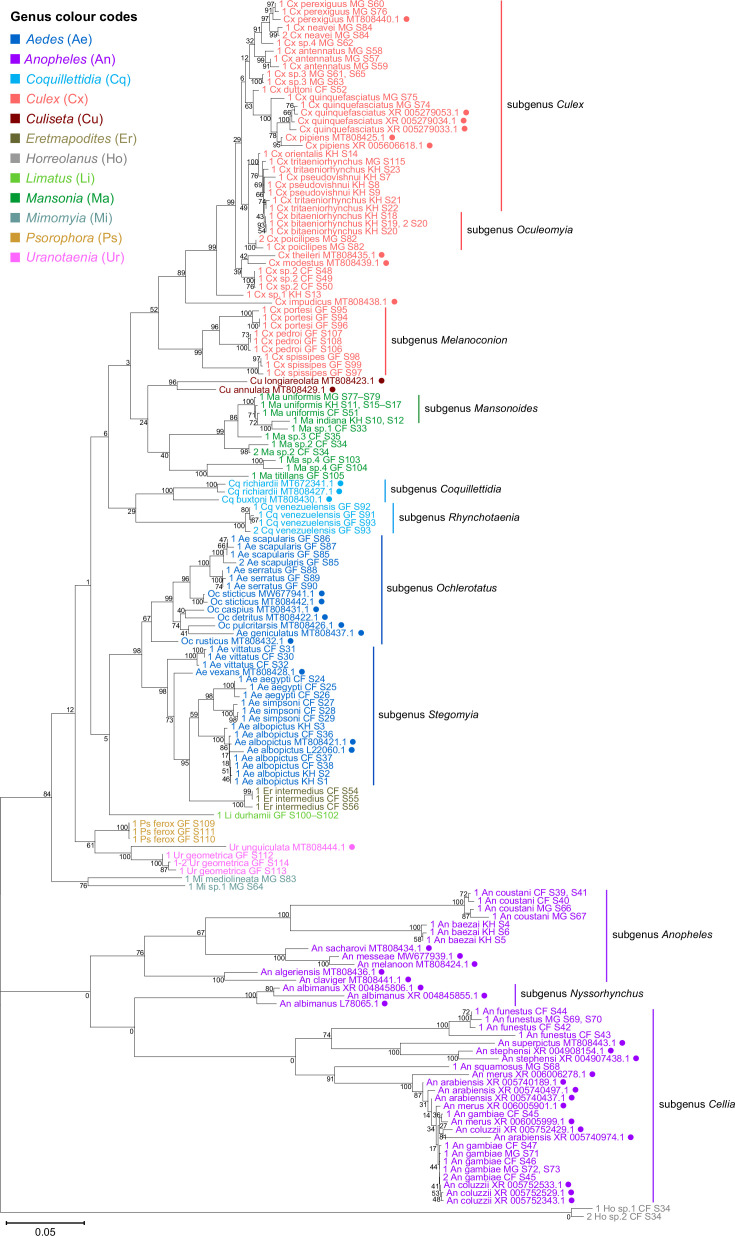
28S sequences generated from this study clustered with conspecifics or congenerics from existing GenBank records. A rooted phylogenetic tree based on full-length 28S sequences (3,900 bp) from this study and from GenBank was inferred using the maximum-likelihood method and constructed to scale in MEGA X (Kumar et al., 2018) using an unknown *Horreolanus* species found among our samples as an outgroup. Values at each node indicate bootstrap support (%) from 500 replications. Sequences from GenBank are annotated with filled circles and their accession numbers are shown. For sequences from this study, each specimen label contains information on taxonomy, origin (in two-letter country codes), and specimen ID number. Some specimens produced up to two consensus 28S sequences; this is indicated by the numbers 1 or 2 at the beginning of the specimen label. Specimen genera are indicated by colour: *Culex* in coral, *Anopheles* in purple, *Aedes* in dark blue, *Mansonia* in dark green, *Culiseta* in maroon, *Limatus* in light green, *Coquillettidia* in light blue, *Psorophora* in yellow, *Mimomyia* in teal, *Uranotaenia* in pink, and *Eretmapodites* in brown. Scale bar at 0.05 is shown. Figure 3—source data 1.Multiple sequence alignment of 169 28S rRNA sequences from this study and from GenBank (FASTA).

To verify the assembly accuracy of our rRNA sequences, we constructed a comprehensive phylogenetic tree from the full-length 28S rRNA sequences generated from our study and included relevant rRNA sequences publicly available from GenBank (Figure 3). We applied a search criterion for GenBank sequences with at least 95% coverage of our sequence lengths (~4000 bp), aiming to represent as many species or genera as possible. Although we rarely found records for the same species included in our study, the resulting tree showed that our 28S sequences generally clustered according to their respective species and subgenera, supported by moderate to good bootstrap support at terminal nodes. Species taxa generally formed monophyletic clades, with the exception of *An. gambiae* and *Cx. quinquefasciatus. An. gambiae* 28S rRNA sequences formed a clade with closely related sequences from *Anopheles arabiensis*, *Anopheles merus*, and *Anopheles coluzzii,* suggesting unusually high interspecies homology for Anophelines or other members of subgenus *Cellia* (Figure 3, in purple, subgenus *Cellia*). Meanwhile, *Cx. quinquefasciatus* 28S rRNA sequences formed a taxon paraphyletic to sister species *Culex pipiens* (Figure 3, in coral, subgenus *Culex*).

**Figure 3—figure supplement 1.**
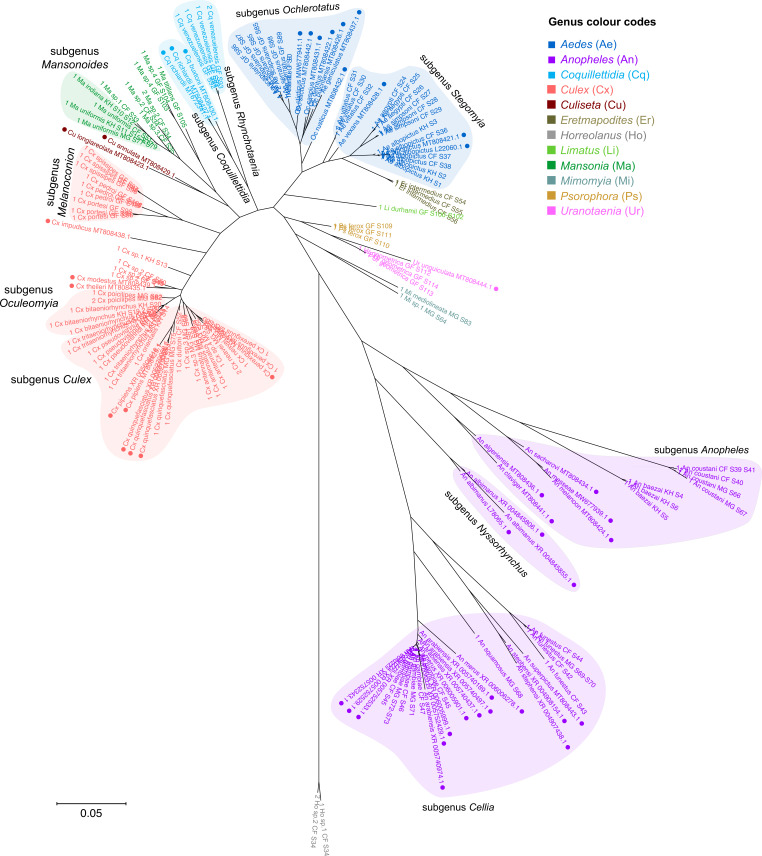
Interspecific and intersubgeneric distances within the genus *Anopheles* indicate a greater degree of divergence than those within any other genera of family Culicidae. The phylogenetic tree presented in Figure 3 based on 28S sequences from this study and from GenBank (annotated with filled circles) is depicted here in radial format to illustrate how the branch lengths separating Anopheline taxa are longer relative to other members of family Culicidae. An unknown *Horreolanus* species found among our samples serves as an outgroup. For sequences from this study, each specimen label contains information on taxonomy, origin (in two-letter country codes), and specimen ID number. Some specimens produced up to two consensus 28S sequences; this is indicated by the numbers 1 or 2 at the beginning of the specimen label. Specimen genera are indicated by colour: *Culex* in coral, *Anopheles* in purple, *Aedes* in dark blue, *Mansonia* in dark green, *Limatus* in light green, *Coquillettidia* in light blue, *Psorophora* in yellow, *Mimomyia* in teal, *Uranotaenia* in pink, and *Eretmapodites* in brown. Scale bar at 0.05 is shown.

**Figure 3—figure supplement 2.**
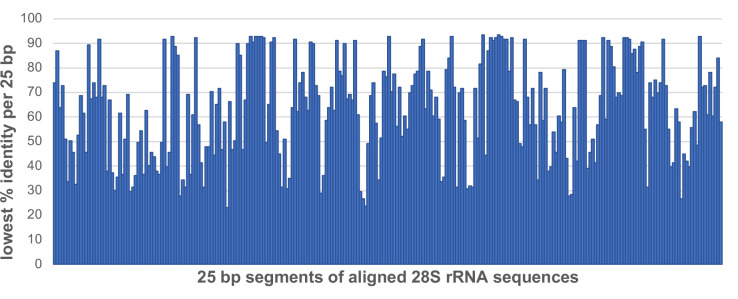
Sequence conservation among 169 28S rRNA sequences obtained from this study and from GenBank combined. Multiple sequence alignment was performed on 28S rRNA sequences, 3,900 bp in length. Each bar represents a 25 bp sliding window of the 28S rRNA sequence alignment where the y-axis values are the lowest percentage nucleotide identity found.

28S rRNA sequence-based phylogenetic reconstructions (Figure 3, with GenBank sequences; Figure 4—figure supplement 1, this study only) showed marked incongruence to that of 18S rRNA sequences (Figure 4—figure supplement 2). Although all rRNA trees show the bifurcation of family Culicidae into subfamilies Anophelinae (genus *Anopheles*, in purple) and Culicinae (all other genera), the recovered intergeneric phylogenetic relationships vary between the 28S and 18S rRNA trees and are weakly supported. The 18S rRNA tree also exhibited several taxonomic anomalies: (i) the lack of definitive clustering by species within the *Culex* subgenus (in coral); (ii) the lack of distinction between 18S rRNA sequences of *Cx. pseudovishnui* and *Cx. tritaeniorhynchus* (in coral); (iii) the placement of Ma sp.3 CF S35 (in dark green) within a *Culex* clade; and (iv) the lack of a monophyletic *Mimomyia* clade (in teal) (Figure 4—figure supplement 2). However, 28S and 18S rRNA sequences are encoded by linked loci in rDNA clusters and should not be analysed separately.

**Figure 4.**
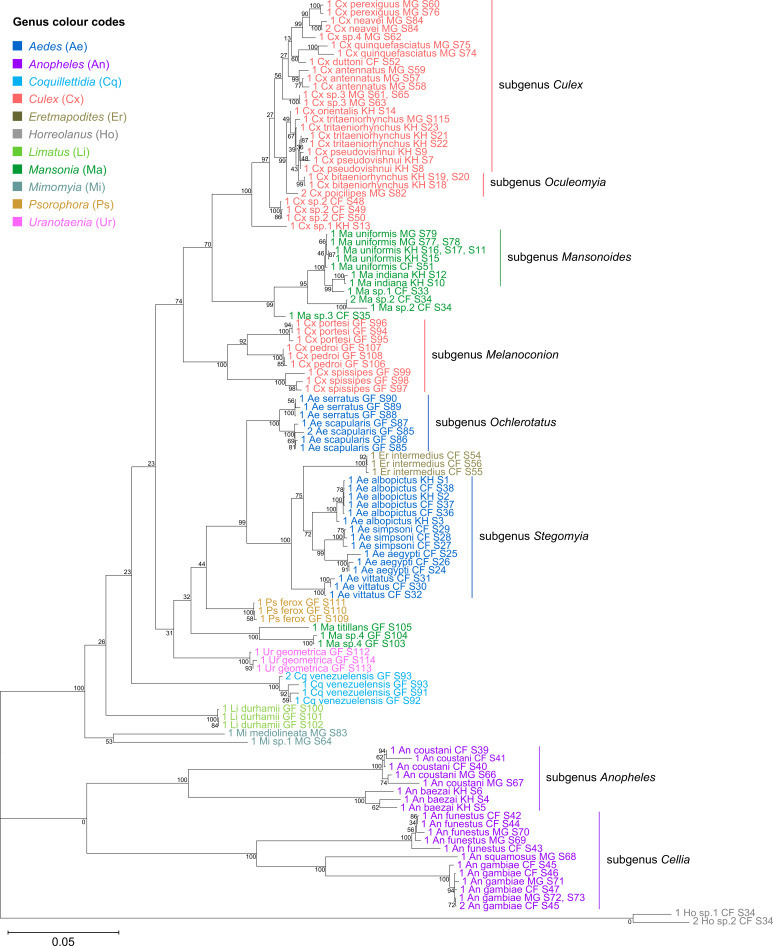
Concatenating 28S and 18S rRNA sequences produces phylogenetic relationships that are concordant with classical Culicidae systematics with higher bootstrap support than 28S sequences alone. This phylogenetic tree based on concatenated 28S+18S rRNA sequences (3,900+1,900 bp) generated from this study was inferred using the maximum-likelihood method and constructed to scale using MEGA X (Kumar et al., 2018) using an unknown *Horreolanus* species found among our samples as an outgroup. Values at each node indicate bootstrap support (%) from 500 replications. Each specimen label contains information on taxonomy, origin (as indicated in two-letter country codes), and specimen ID number. Some specimens produced up to two consensus 28S+18S rRNA sequences; this is indicated by the numbers 1 or 2 at the beginning of the specimen label. Specimen genera are indicated by colour: *Culex* in coral, *Anopheles* in purple, *Aedes* in dark blue, *Mansonia* in dark green, *Limatus* in light green, *Coquillettidia* in light blue, *Psorophora* in yellow, *Mimomyia* in teal, *Uranotaenia* in pink, and *Eretmapodites* in brown. Scale bar at 0.05 is shown. Figure 4—source data 1.Multiple sequence alignment of 122 28S rRNA sequences, including two sequences from *Horreolanus* sp. (FASTA). Figure 4—source data 2.Multiple sequence alignment of 114 18S rRNA sequences, including two sequences from *Horreolanus* sp. (FASTA).

Indeed, when concatenated 28S+18S rRNA sequences were generated from the same specimens (Figure 4), the phylogenetic tree resulting from these sequences more closely resembles the 28S tree (Figure 3) with regard to the basal position of the *Mimomyia* clade (in teal) within the Culicinae subfamily with good bootstrap support in either tree (84% in 28S rRNA tree, 100% in concatenated 28S+18S rRNA tree). For internal nodes, bootstrap support values were higher in the concatenated tree compared to the 28S tree. Interestingly, the 28S+18S rRNA tree formed an *Aedini* tribe-clade encompassing taxa from genera *Psorophora* (in yellow)*, Aedes* (in dark blue)*,* and *Eretmapodites* (in brown), possibly driven by the inclusion of 18S rRNA sequences. Concatenation also resolved the anomalies found in the 18S rRNA tree and added clarity to the close relationship between *Culex* (in coral) and *Mansonia* (in dark green) taxa. Of note, relative to the 28S tree (Figure 3) the *Culex* and *Mansonia* genera are no longer monophyletic in the concatenated 28S+18S rRNA tree (Figure 4). Genus *Culex* is paraphyletic with respect to subgenus *Mansonoides* of genus *Mansonia* (Figure 3). *Ma. titillans* and Ma sp.4, which we suspect to be *Mansonia pseudotitillans,* always formed a distinct branch in 28S or 18S rRNA phylogenies, thus possibly representing a clade of subgenus *Mansonia*.

**Figure 4—figure supplement 1.**
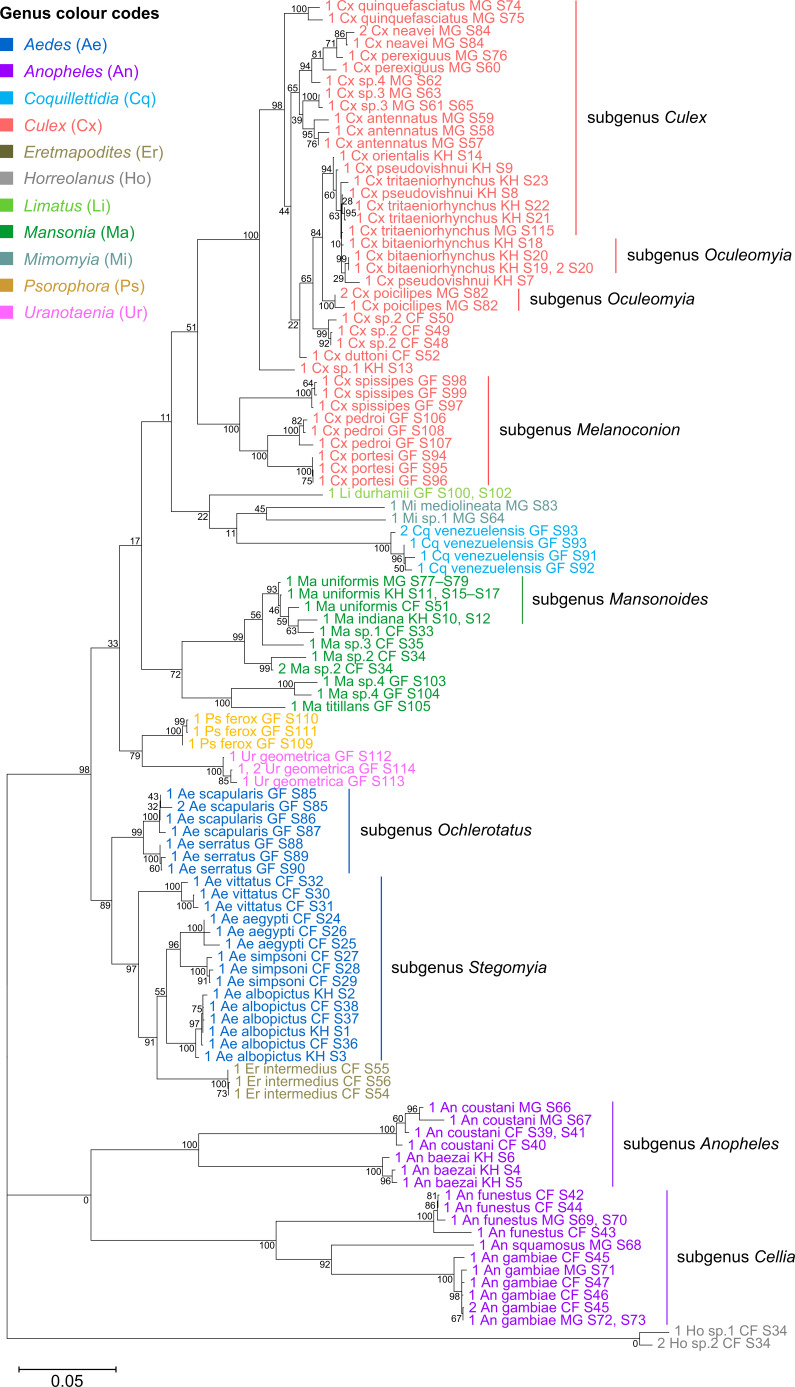
Phylogenetic tree based on 28S rRNA sequences generated from this study (3,900 bp). This tree was inferred using maximum-likelihood method and constructed to scale in MEGA X (Kumar et al., 2018) using an unknown *Horreolanus* species found among our samples as an outgroup. Values at each node indicate bootstrap support (%) from 500 replications. For sequences from this study, each specimen label contains information on taxonomy, origin (in two-letter country codes), and specimen ID number. Some specimens produced up to two consensus 28S rRNA sequences; this is indicated by the numbers 1 or 2 at the beginning of the specimen label. Specimen genera are indicated by colour: *Culex* in coral, *Anopheles* in purple, *Aedes* in dark blue, *Mansonia* in dark green, *Limatus* in light green, *Coquillettidia* in light blue, *Psorophora* in yellow, *Mimomyia* in teal, *Uranotaenia* in pink, and *Eretmapodites* in brown. Scale bar at 0.05 is shown.

**Figure 4—figure supplement 2.**
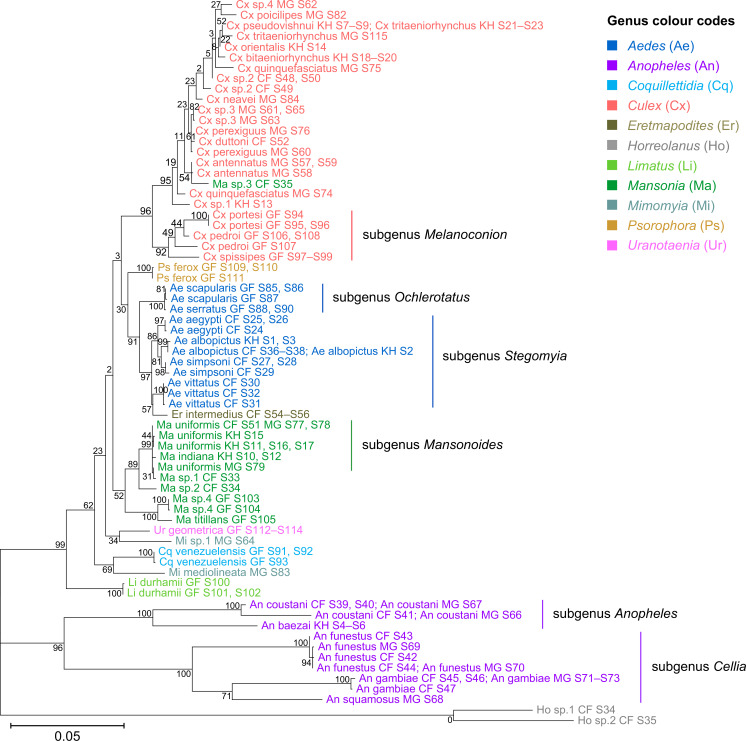
Phylogenetic tree based on 18S rRNA sequences (1,900 bp). This tree was inferred using maximum-likelihood method and constructed to scale in MEGA X (Kumar et al., 2018) using an unknown *Horreolanus* species found among our samples as an outgroup. Values at each node indicate bootstrap support (%) from 500 replications. For sequences from this study, each specimen label contains information on taxonomy, origin (in two-letter country codes), and specimen ID number. One 18S rRNA sequence was obtain for each specimen. Specimen genera are indicated by colour: *Culex* in coral, *Anopheles* in purple, *Aedes* in dark blue, *Mansonia* in dark green, *Limatus* in light green, *Coquillettidia* in light blue, *Psorophora* in yellow, *Mimomyia* in teal, *Uranotaenia* in pink, and *Eretmapodites* in brown. Scale bar at 0.05 is shown.

The concatenated 28S+18S rRNA tree (Figure 4) recapitulates what is classically known about the systematics of our specimens, namely (i) the early divergence of subfamily Anophelinae from subfamily Culicinae, (ii) the division of genus *Anopheles* (in purple) into two subgenera, *Anopheles* and *Cellia,* (iii) the division of genus *Aedes* (in dark blue) into subgenera *Stegomyia* and *Ochlerotatus*, (iv) the divergence of the monophyletic subgenus *Melanoconion* within the *Culex* genus (in coral) (Harbach, 2007; Harbach and Kitching, 2016).

### rRNA as a molecular marker for taxonomy and phylogeny

We sequenced a 621 bp region of the COI gene to confirm morphological species identification of our specimens and to compare the functionality of rRNA and COI sequences as molecular markers for taxonomic and phylogenetic investigations. COI sequences were able to unequivocally determine the species identity in most specimens except for the following cases. *An. coustani* COI sequences from our study, regardless of specimen origin, shared remarkably high nucleotide similarity (>98%) with several other *Anopheles* species such as *An. rhodesiensis*, *An. rufipes*, *An. ziemanni*, *An. tenebrosus*, although *An. coustani* remained the most frequent and closest match. In the case of *Ae. simpsoni*, three specimens had been morphologically identified as *Ae. opok* although their COI sequences showed 97–100% similarity to that of *Ae. simpsoni*. As GenBank held no records of *Ae. opok* COI at the time of this study, we instead aligned the putative *Ae. simpsoni* COI sequences against two sister species of *Ae. opok: Ae. luteocephalus* and *Ae. africanus*. We found they shared only 90% and 89% similarity, respectively. Given this significant divergence, we concluded these specimens to be *Ae. simpsoni*. Ambiguous results were especially frequent among *Culex* specimens belonging to the *Cx. pipiens* or *Cx. vishnui* subgroups, where the query sequence differed with either of the top two hits by a single nucleotide. For example, between *Cx. quinquefasciatus* and *Cx. pipiens* of the *Cx. pipiens* subgroup, and between *Cx. vishnui* and *Cx. tritaeniorhynchus* of the *Cx. vishnui* subgroup.

Among our three specimens of *Ma. titillans*, two appeared to belong to a single species that is different from but closely related to *Ma. titillans*. We surmised that these specimens could instead be *Ma. pseudotitillans* based on morphological similarity but were not able to verify this by molecular means as no COI reference sequence is available for this species. These specimens are hence putatively labelled as ‘Ma sp.4’.

**Figure 5.**
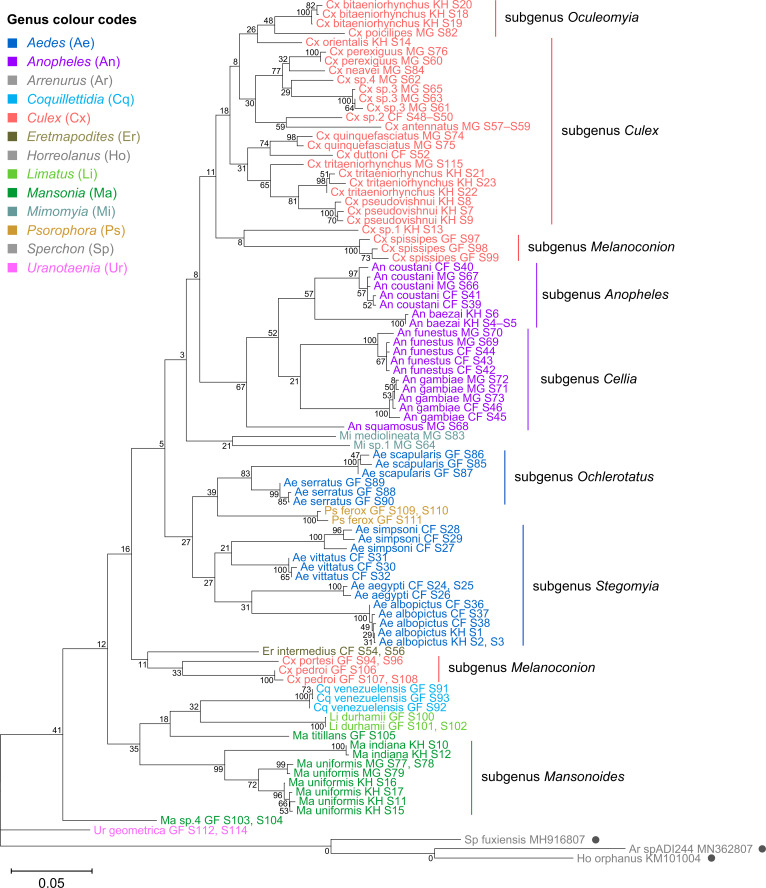
Cytochrome *c* oxidase I (COI) sequences cluster by species but show phylogenetic relationships that contrast those derived from rRNA trees. A phylogenetic tree based on COI sequences (621–699 bp) was inferred using the maximum-likelihood method and constructed to scale using MEGA X (Kumar et al., 2018) with three water mite species to serve as outgroups. Outgroup sequences obtained from GenBank are annotated with filled circles and their accession numbers are shown. Values at each node indicate bootstrap support (%) from 500 replications. Each specimen label contains information on taxonomy, origin (as indicated in two-letter country codes), and specimen ID. Specimen genera are indicted by colour: *Culex* in coral, *Anopheles* in purple, *Aedes* in dark blue, *Mansonia* in dark green, *Limatus* in light green, *Coquillettidia* in light blue, *Psorophora* in yellow, *Mimomyia* in teal, *Uranotaenia* in pink, and *Eretmapodites* in brown. Scale bar at 0.05 is shown. Figure 5—source data 1.Multiple sequence alignment of 106 cytochrome *c* oxidase I (COI) sequences (FASTA).

Phylogenetic reconstruction based on the COI sequences showed clustering of all species taxa into distinct clades, underlining the utility of the COI gene in molecular taxonomy (Figure 5; Hebert et al., 2003; Ratnasingham and Hebert, 2007). However, species delineation among members of *Culex* subgroups were not as clear-cut, although sister species were correctly placed as sister taxa (Figure 5, in coral). This is comparable to the 28S+18S rRNA tree (Figure 4, in coral) and is indicative of lower intraspecies distances relative to interspecies distances.

To evaluate the utility of 28S and 18S rRNA sequences for molecular taxonomy, we used the 28S+18S rRNA tree to discern the identity of six specimens for which COI sequencing could not be performed. These specimens include three unknown *Mansonia* species (Specimen ID S33–S35), a *Ma. uniformis* (Specimen ID S51), an *An. gambiae* (Specimen ID S47), and a *Ur. geometrica* (Specimen ID S113) (Appendix 1—table 1). Their positions in the 28S+18S rRNA tree relative to adjacent taxa confirms the morphological identification of all six specimens to the genus level and, for three of them, to the species level (Figure 4; *Mansonia* in dark green, *Anopheles* in purple, *Uranotaenia* in pink).

The phylogenetic relationships indicated by the COI tree compared to the 28S+18S rRNA tree present only few points of similarity, with key differences summarised in Table 2. COI-based phylogenetic inference indeed showed clustering of generic taxa into monophyletic clades albeit with very weak bootstrap support, except for genera *Culex* and *Mansonia* (Figure 5; *Culex* in coral, *Mansonia* in dark green). Contrary to the 28S+18S rRNA tree (Figure 4), *Culex* subgenus *Melanoconion* was depicted as a polyphyletic taxon with *Cx. spissipes* being a part of the greater *Culicini* clade with members from subgenera *Oculeomyia* and *Culex* while *Cx. pedroi* and *Cx. portesi* formed a distantly related clade. Among the *Mansonia* specimens, the two unknown Ma sp.4 specimens were not positioned as the nearest neighbours of *Ma. titillans* and instead appeared to have diverged earlier from most of the other taxa from the *Culicidae* family. Notably, the COI sequences of genus *Anopheles* (Figure 5, in purple) is not basal to the other members of *Culicidae* and is instead shown to be sister to *Culex* COI sequences (8% bootstrap support). This is a direct contrast to what is suggested by the rRNA phylogenies (Figures 3 and 4, Figure 4—figure supplements 1 and 2; *Anopheles* in purple), which suggests *Culex* (in coral) rRNA sequences to be among the most recently diverged. Bootstrap support for the more internal nodes of the COI trees were remarkably low compared to those of rRNA-based trees.

**Table 2.** Summary of differences between rRNA and cytochrome *c* oxidase I (COI) phylogenies.

Taxa	28S+18S rRNA phylogeny (Figure 4)	COI phylogeny (Figure 5)
The *Anopheles* genus	Forms a clade that is basal to the all other members of family Culicidae; interspecies branch lengths are notably long	Forms a sister clade to the *Culex* genus, and is depicted to have diverged more recently; interspecies branch lengths are comparable to that of other genera
The *Ur. geometrica* species	Forms a clade within the Culicinae subfamily lineage	Forms a clade that is basal to the all other members of family Culicidae
*The Aedini tribe*	Forms a monophyletic clade comprising the genera *Aedes, Eretmapodites,* and *Psorophora*, with the latter being an early divergent lineage	Does not form a monophyletic clade; the *Psorophora* clade is placed among *Aedes* taxa and the *Eretmapodites* clade is sister to a *Culex* subgenus *Melanoconion* clade
The *Culex* genus	Splits into two monophyletic clades with the three French Guyanese species forming a closely related minor clade	Splits into two clades with two out of three French Guyanese species (*Cx. pedroi* and *Cx. portesi*) forming a distantly related minor clade, while the third (*Cx. spissipes*) is a part of the greater clade
The *Mansonia* genus	Is a polyphyletic group comprising two clades with the two French Guyanese taxa forming a distantly related minor clade; the major clade is placed among *Culex* taxa	Forms a subgenus *Mansonoides* clade as per the 28S+18S rRNA tree but the French Guyanese taxa do not cluster together; is depicted to have diverged earlier relative to other taxa in the assemblage
The Ma sp.4 species	Forms a sister clade to *Ma. titillans* as part of a minor French Guyanese *Mansonia* clade	Does not form a sister clade to *Ma. titillans;* instead is shown to have diverged earlier than all other members of family Culicidae after *Ur. geometrica*

In all rRNA trees, it is clear that the interspecific and intersubgeneric evolutionary distances within the genus *Anopheles* are high relative to any other genera, indicating a greater degree of divergence (Figure 3, Figure 3—figure supplement 1, Figure 4, Figure 4—figure supplements 1 and 2; *Anopheles* in purple). This is evidenced by the longer branch lengths connecting Anopheline species-clades to the node of the most recent common ancestor for subgenera *Anopheles* and *Cellia*. This feature is not evident in the COI tree, where the Anopheline interspecies distances are comparable to those within the *Culex*, *Aedes*, and *Mansonia* taxa (Figure 5; *Anopheles* in purple, *Culex* in coral, *Aedes* in dark blue, *Mansonia* in dark green).

### On *Culex* subgroups

*Culex* (subgenus *Culex*) specimens of this study comprise several closely related sister species belonging to the *Cx. vishnui* and *Culex univittatus* subgroups, which are notoriously difficult to differentiate based on morphology. Accordingly, in the 28S+18S rRNA (Figure 4, in coral) and COI (Figure 5, in coral) trees these species and their known sister species were clustered together within the *Culex* (subgenus *Culex*) clade: *Cx. tritaeniorhynchus* with *Cx. pseudovishnui* (*Cx. vishnui* subgroup); *Cx. perexiguus* with *Cx. neavei* (*Cx. univittatus* subgroup).

The use of the COI sequence to distinguish between members of the *Culex* subgroups was limited. For example, for the two *Cx. quinquefasciatus* samples in our taxonomic assemblage (Specimen ID S74 and S75) (Appendix 1—table 1), BLAST analyses of their COI sequences revealed they are a single nucleotide apart from *Cx. pipiens* or *Cx. quinquefasciatus* COI sequences (Appendix 2—table 1). In the 28S rRNA tree with GenBank sequences (Figure 3), two *Cx. pipiens* GenBank sequences formed a clade sister to another containing three *Cx. quinquefasciatus* GenBank sequences and the ‘Cx quinquefasciatus MG S74’ sequence with 78% bootstrap support. This is in accordance with other studies examining mitochondrial sequences (Sun et al., 2019) and morphological attributes (Harbach et al., 2017). This shows that the 28S rRNA sequence can distinguish the two species and confirms that ‘Cx quinquefasciatus MG S74’ is indeed a *Cx. quinquefasciatus* specimen. However, ‘Cx quinquefasciatus MG S75’ is shown to be basal from other sequences within this *Cx. pipiens* subgroup-clade with 100% bootstrap support. Given that *Cx. quinquefasciatus* and *Cx. pipiens* are known to interbreed, it is plausible that this individual is a hybrid of the two species (Farajollahi et al., 2011).

## Discussion

RNA-seq metagenomics on field-captured sylvatic mosquitoes is a valuable tool for tracking mosquito viruses through surveillance and virus discovery. However, the lack of reference rRNA sequences hinders good oligo-based depletion and efficient clean-up of RNA-seq data. Additionally, de novo assembly of rRNA sequences is complicated due to regions that are highly conserved across all distantly related organisms that could be present in a single specimen, that is, microbiota, parasites, or vertebrate blood meal. Hence, we established a method to bioinformatically filter out non-host rRNA reads for the accurate assembly of novel 28S and 18S rRNA reference sequences.

We found that phylogenetic reconstructions based on 28S sequences or concatenated 28S+18S rRNA sequences were able to correctly cluster mosquito taxa according to species and corroborate current mosquito classification. This demonstrates that our bioinformatics methodology reliably generates bona fide 28S and 18S rRNA sequences, even in specimens parasitized by water mites or engorged with vertebrate blood. Further, we were able to use 28S+18S rRNA sequence taxonomy for molecular species identification when COI sequences were unavailable or ambiguous, thus supporting the use of rRNA sequences as a molecular marker. In RNA-seq metagenomics applications, they have the advantage of circumventing the need to additionally isolate and sequence DNA from specimens, as RNA-seq reads can be directly mapped against reference sequences. In our hands, there are sufficient numbers of remaining reads post-depletion (5–10% of reads per sample) to assemble complete rRNA contigs (unpublished data).

Phylogenetic inferences based on 28S or 18S rRNA sequences alone do not recover the same interspecific relationships (Figure 4—figure supplements 1 and 2). Relative to 28S sequences, we observed more instances where multiple specimens have near-identical 18S rRNA sequences. This can occur for specimens belonging to the same species, but also for conspecifics sampled from different geographic locations, such as *An. coustani*, *An. gambiae*, or *Ae. albopictus*. More rarely, specimens from the same species subgroup, such as *Cx. pseudovishnui* and *Cx. tritaeniorhynchus*, also shared 18S rRNA sequences. This was surprising given that the 18S rRNA sequences in our dataset is 1,900 bp long. Concatenation of 28S and 18S rRNA sequences resolved this issue, enabling species delineation even among sister species of *Culex* subgroups, where morphological identification meets its limits.

In Cambodia and other parts of Asia, the *Cx. vishnui* subgroup includes *Cx. tritaeniorhynchus*, *Cx. vishnui*, and *Cx. pseudovishnui*, which are important vectors of JEV (Maquart and Boyer, 2022). The former two were morphologically identified in our study but later revealed by COI sequencing to be a sister species. Discerning sister species of the *Cx. pipiens* subgroup is further complicated by interspecific breeding, with some populations showing genetic introgression to varying extents (Cornel et al., 2003). The seven sister species of this subgroup are practically indistinguishable based on morphology and require molecular methods to discern (Farajollahi et al., 2011; Zittra et al., 2016). Indeed, the 621 bp COI sequence amplified in our study did not contain enough nucleotide divergence to allow clear identification, given that the COI sequence of *Cx. quinquefasciatus* specimens differed from that of *Cx. pipiens* by a single nucleotide. Batovska et al., 2017, found that even the Internal Transcribed Spacer 2 (ITS2) rDNA region, another common molecular marker, could not differentiate the two species. Other DNA molecular markers such as nuclear Ace-2 or CQ11 genes (Aspen and Savage, 2003; Zittra et al., 2016) or *Wolbachia pipientis* infection status (Cornel et al., 2003) are typically employed in tandem. In our study, 28S rRNA sequence-based phylogeny validated the identity of specimen ‘Cx quinquefasciatus MG S74’ (Figure 3, in coral) and suggested that specimen ‘Cx quinquefasciatus MG S75’ might have been a *pipiens-quinquefasciatus* hybrid. These examples demonstrate how 28S rRNA sequences, concatenated with 18S rRNA sequences or alone, contain enough resolution to differentiate between *Cx. pipiens* and *Cx. quinquefasciatus*. rRNA-based phylogeny thus allows for more accurate species identification and ecological observations in the context of disease transmission. Additionally, tracing the genetic flow across hybrid populations within the *Cx. pipiens* subgroup can inform estimates of vectorial capacity for each species. As only one or two members from the *Cx. pipiens* and *Cx. vishnui* subgroups were represented in our taxonomic assemblage, an explicit investigation including all member species of these subgroups in greater sample numbers is warranted to further test the degree of accuracy with which 28S and 18S rRNA sequences can delineate sister species.

Our study included French Guianese *Culex* species *Cx. spissipes* (group Spissipes), *Cx. pedroi* (group Pedroi), and *Cx. portesi* (group Vomerifer). These species belong to the New World subgenus *Melanoconion*, section Spissipes, with well-documented distribution in North and South Americas (Sirivanakarn, 1982) and are vectors of encephalitic alphaviruses EEEV and VEEV among others (Talaga et al., 2021; Turell et al., 2008; Weaver et al., 2004). Indeed, our rooted rRNA and COI trees showed the divergence of the three *Melanoconion* species from the major *Culex* clade comprising species broadly found across Africa and Asia (Auerswald et al., 2021; Farajollahi et al., 2011; Nchoutpouen et al., 2019; Takhampunya et al., 2011). The topology of the concatenated 28S+18S rRNA tree places the *Cx. portesi* and *Cx. pedroi* species-clades as sister groups (92% bootstrap support), with *Cx. spissipes* as a basal group within the *Melanoconion* clade (100% bootstrap support) (Figure 4, in coral). This corroborates the systematics elucidated by Navarro and Weaver, 2004, using the ITS2 marker, and those by Sirivanakarn, 1982 and Sallum and Forattini, 1996 based on morphology. Curiously, in the COI tree, *Cx. spissipes* sequences were clustered with unknown species Cx. sp.1, forming a clade sister to another containing other *Culex* (*Culex*) and *Culex* (*Oculeomyia*) species, albeit with very low bootstrap support (Figure 5, in coral). Previous phylogenetic studies based on the COI gene have consistently placed *Cx. spissipes* or the Spissipes group basal to other groups within the *Melanoconion* subgenus (Torres-Gutierrez et al., 2016; Torres-Gutierrez et al., 2018). However, these studies contain only *Culex* (*Melanoconion*) species in their assemblage, apart from *Cx. quinquefasciatus* to act as an outgroup. This clustering of *Cx. spissipes* with non-*Melanoconion* species in our COI phylogeny could be an artefact of a much more diversified assemblage rather than a true phylogenetic link.

Taking advantage of our multi-country sampling, we examined whether rRNA or COI phylogeny can be used to distinguish conspecifics originating from different geographies. Our assemblage contains five of such species: *An. coustani*, *An. funestus*, *An. gambiae*, *Ae. albopictus*, and *Ma. uniformis*. Among the rRNA trees, the concatenated 28S+18S and 28S rRNA trees were able to discriminate between *Ma. uniformis* specimens from Madagascar, Cambodia, and the Central African Republic (in dark green), and between *An. coustani* specimens from Madagascar and the Central African Republic (in purple) (100% bootstrap support). In the COI tree, only *Ma. uniformis* was resolved into geographical clades comprising specimens from Madagascar and specimens from Cambodia (in dark green) (72% bootstrap support). No COI sequence was obtained from one *Ma. uniformis* specimen from the Central African Republic. The 28S+18S rRNA sequences ostensibly provided more population-level genetic information than COI sequences alone with better support. The use of rRNA sequences in investigating the biodiversity of mosquitoes should therefore be explored with a more comprehensive taxonomic assemblage.

The phylogenetic reconstructions based on rRNA or COI sequences in our study are hardly congruent (Table 2), but two principal differences stand out. First, the COI phylogeny does not recapitulate the early divergence of Anophelinae from Culicinae (Figure 5). This is at odds with other studies estimating mosquito divergence times based on mitochondrial genes (Logue et al., 2013; Lorenz et al., 2021) or nuclear genes (Reidenbach et al., 2009). The second notable feature in the rRNA trees is the remarkably large interspecies and intersubgeneric evolutionary distances within genus *Anopheles* relative to other genera in the Culicinae subfamily (Figure 3, Figure 3—figure supplement 1, Figure 4, Figure 4—figure supplements 1 and 2; *Anopheles* in purple) but this is not apparent in the COI tree. The hyperdiversity among *Anopheles* taxa may be attributed to the earlier diversification of the Anophelinae subfamily in the early Cretaceous period compared to that of the Culicinae subfamily—a difference of at least 40 million years (Lorenz et al., 2021). The differences in rRNA and COI tree topologies indicate a limitation in using COI alone to determine evolutionary relationships. Importantly, drawing phylogenetic conclusions from short DNA markers such as COI has been cautioned against due to its weak phylogenetic signal (Hajibabaei et al., 2006). The relatively short length of our COI sequences (621–699 bp) combined with the 100-fold higher nuclear substitution rate of mitochondrial genomes relative to nuclear genomes (Arctander, 1995) could result in homoplasy (Danforth et al., 2005), making it difficult to clearly discern ancestral sequences and correctly assign branches into lineages, as evidenced by the poor nodal bootstrap support at genus-level branches. Indeed, in the study by Lorenz et al., 2021, a phylogenetic tree constructed using a concatenation of all 13 protein-coding genes of the mitochondrial genome was able to resolve ancient divergence events. This affirms that while COI sequences can be used to reveal recent speciation events, longer or multi-gene molecular markers are necessary for studies into deeper evolutionary relationships (Danforth et al., 2005).

In contrast to Anophelines where 28S rRNA phylogenies illustrated higher interspecies divergence compared to COI phylogeny, two specimens of an unknown *Mansonia* species, ‘Ma sp.4 GF S103’ and ‘Ma sp.4 GF S104’, provided an example where interspecies relatedness based on their COI sequences is greater than that based on their rRNA sequences in relation to ‘Ma titillans GF S105’. While all rRNA trees placed ‘Ma titillans GF S105’ as a sister taxon with 100% bootstrap support, the COI tree placed M sp.4 basal to all other species except *Ur. geometrica* (Figure 5; *Mansonia* in dark green, *Uranotaenia* in pink). This may hint at a historical selective sweep in the mitochondrial genome, whether arising from geographical separation, mutations, or linkage disequilibrium with inherited symbionts (Hurst and Jiggins, 2005), resulting in the disparate mitochondrial haplogroups found in French Guyanese Ma sp.4 and *Ma. titillans*. In addition, both haplogroups are distant from those associated with members of subgenus *Mansonoides*. To note, the COI sequences of ‘M sp.4 GF S103’ and ‘M sp.4 GF S104’ share 87.12% and 87.39% nucleotide similarity, respectively, to that of ‘Ma titillans GF S105’. Interestingly, the endosymbiont *Wo. pipientis* has been detected in *Ma. titillans* sampled from Brazil (de Oliveira et al., 2015), which may contribute to the divergence of ‘Ma titillans GF S105’ COI sequence away from those of Ma sp.4. This highlights other caveats of using a mitochondrial DNA marker in determining evolutionary relationships (Hurst and Jiggins, 2005), which nuclear markers such as 28S and 18S rRNA sequences may be immune to.

### Conclusions

Total RNA-seq is a valuable tool for surveillance and virus discovery in sylvatic mosquitoes but it is impeded by the lack of full-length rRNA reference sequences. Here, we presented an rRNA sequence assembly strategy and a dataset of 234 newly generated mosquito 28S and 18S rRNA sequences. Our work has expanded the current mosquito rRNA reference library by providing, to our knowledge, the first full-length rRNA records for 30 species in public databases and paves the way for the assembly of many more. These novel rRNA sequences can improve mosquito metagenomics based on RNA-seq by enabling physical and computational removal of rRNA from specimens and streamlined species identification using rRNA markers.

Given that a reference sequence is available, rRNA markers could serve as a better approach for mosquito taxonomy and phylogeny than COI markers. In analysing the same set of specimens based on their COI and rRNA sequences, we showed that rRNA sequences can discriminate between members of a species subgroup as well as conspecifics from different geographies. Phylogenetic inferences from a tree based on 28S rRNA sequences alone or on concatenated 28S+18S rRNA sequences are more aligned with contemporary mosquito systematics, showing evolutionary relationships that agree with other phylogenetic studies. While COI-based phylogeny can reveal recent speciation events, rRNA sequences may be better suited for investigations of deeper evolutionary relationships as they are less prone to selective sweeps and homoplasy. The advantages and disadvantages of rRNA and COI sequences as molecular markers are summarised in Table 3. Further studies are necessary to reveal how rRNA sequences compare against other nuclear or mitochondrial DNA marker systems (Batovska et al., 2017; Beebe, 2018; Behura, 2006; Ratnasingham and Hebert, 2007; Reidenbach et al., 2009; Vezenegho et al., 2022).

**Table 3.** Comparison of 28S or concatenated 28S+18S rRNA and cytochrome *c* oxidase I (COI) sequences as molecular markers.

28S+18S rRNA	
Advantages	Disadvantages
In RNA-seq metagenomics studies, molecular taxonomy of specimens based on rRNA sequences can be done from RNA-seq data without additional sample preparation or sequencing. 28S rRNA and concatenated 28S+18S rRNA sequences can resolve the identity of specimens where COI sequences were ambiguous, particularly between members of species subgroups. 28S rRNA and concatenated 28S+18S rRNA sequences can distinguish conspecifics from different geographies for certain species. Phylogenetic inferences based on 28S rRNA and concatenated 28S+18S rRNA sequences show relationships that are more concordant to contemporary mosquito systematics elucidated by other studies and may be a more suitable marker to study deep evolutionary relationships. Being longer and nuclear-encoded, 28S or concatenated 28S+18S rRNA sequences are immune to homoplasy or to selective sweeps that may affect genomes of inherited symbionts such as mitochondria.	RNA-seq costs more than Sanger sequencing. Reference rRNA sequences are currently much more limited in breadth compared to other established molecular markers.
COI	
Advantages	Disadvantages
With a larger reference database, the COI is a versatile marker for molecular taxonomy. Being a shorter DNA marker, the COI gene is cost- and time-effective to amplify, sequence, and characterise. Universal primer sets to amplify the COI marker have been developed and tested for many diverse species.	All species taxa clustered into distinct clades but with weaker bootstrap support at internal nodes relative to those of the 28S+18S rRNA tree. For *An. coustani*, and members of *Culex* species subgroups such as *Cx. quinquefasciatus* and *Cx. tritaeniorhynchus*, COI sequences are unable to unequivocally confirm species identity as species can differ by just one nucleotide. Other molecular markers are often used in tandem.

## Materials and methods

### Sample collection

Mosquito specimens were sampled from 2019 to 2020 by medical entomology teams from the Institut Pasteur de Bangui (Central African Republic, Africa; CF), Institut Pasteur de Madagascar (Madagascar, Africa; MG), Institut Pasteur du Cambodge (Cambodia, Asia; KH), and Institut Pasteur de la Guyane (French Guiana, South America; GF). Adult mosquitoes were sampled using several techniques including CDC light traps, BG sentinels, and human-landing catches. Sampling sites are sylvatic locations including rural settlements in the Central African Republic, Madagascar, and French Guiana and national parks in Cambodia. Mosquitoes were morphologically identified using taxonomic identification keys (Edwards, 1941; Grjebine, 1966; Huang and Ward, 1981; Oo et al., 2006; Rattanarithikul et al., 2007; Rattanarithikul et al., 2010; Rattanarithikul et al., 2005a; Rattanarithikul et al., 2005b; Rattanarithikul et al., 2006a; Rattanarithikul et al., 2006b; Rueda, 2004) on cold tables before preservation by flash freezing in liquid nitrogen and transportation in dry ice to Institut Pasteur Paris for analysis. A list of the 112 mosquito specimens included in our taxonomic assemblage and their related information are provided in Appendix 1—table 1. To note, specimen ID S53, S80, and S81 were removed from our assemblage as their species identity could not be determined by COI or rRNA sequences.

### RNA and DNA isolation

Nucleic acids were isolated from mosquito specimens using TRIzol reagent according to the manufacturer’s protocol (Invitrogen, Thermo Fisher Scientific, Waltham, MA, USA). Single mosquitoes were homogenised into 200 µL of TRIzol reagent and other of the reagents within the protocol were volume-adjusted accordingly. Following phase separation, RNA were isolated from the aqueous phase while DNA were isolated from the remaining interphase and phenol-chloroform phase. From here, RNA is used to prepare cDNA libraries for next-generation sequencing while DNA is used in PCR amplification and Sanger sequencing of the mitochondrial COI gene as further described below.

### Probe depletion of rRNA

We tested a selective rRNA depletion protocol by Morlan et al., 2012 on several mosquito species from the *Aedes*, *Culex*, and *Anopheles* genera. We designed 77 tiled 80 bp DNA probes antisense to the *Ae. aegypti* 28S, 18S, and 5.8S rRNA sequences. A pool of probes at a concentration of 0.04 µM were prepared. To bind probes to rRNA, 1 µL of probes and 2 µL of Hybridisation Buffer (100 mM Tris-HCl and 200 mM NaCl) were added to rRNA samples to a final volume of 20 µL and subjected to a slow-cool incubation starting at 95°C for 2 min, then cooling to 22°C at a rate of 0.1°C per second, ending with an additional 5 min at 22°C. The resulting RNA:DNA hybrids were treated with 2.5 µL Hybridase Thermostable RNase H (Epicentre, Illumina, Madison, WI, USA) and incubated at 37°C for 30 min. To remove DNA probes, the mix was treated with 1 µL DNase I (Invitrogen) and purified with Agencourt RNAClean XP Beads (Beckman Coulter, Brea, CA, USA). The resulting RNA is used for total RNA-seq to check depletion efficiency.

### Total RNA-seq

To obtain rRNA sequences, RNA samples were quantified on a Qubit Fluorometer (Invitrogen) using the Qubit RNA BR Assay kit (Invitrogen) for concentration adjustment. Non-depleted total RNA was used for library preparation for next-generation sequencing using the NEBNext Ultra II RNA Library Preparation Kit for Illumina (New England Biolabs, Ipswich, MA, USA) and the NEBNext Multiplex Oligos for Illumina (Dual Index Primers Set 1) (New England Biolabs). Sequencing was performed on a NextSeq500 sequencing system (Illumina, San Diego, CA, USA). Quality control of fastq data and trimming of adapters were performed with FastQC and cutadapt, respectively.

### 28S and 18S rRNA assembly

To obtain 28S and 18S rRNA contigs, we had to first clean our fastq library by separating the reads representing mosquito rRNA from all other reads. To achieve this, we used the SILVA RNA sequence database to create two libraries: one containing all rRNA sequences recorded under the ‘Insecta’ node of the taxonomic tree, the other containing the rRNA sequences of many other nodes distributed throughout the taxonomic tree, hence named ‘Non-Insecta’ (Quast et al., 2013). Each read was aligned using the nucleotide Basic Local Alignment Search Tool (BLASTn, https://blast.ncbi.nlm.nih.gov/) of the National Center for Biotechnology Information (NCBI) against each of the two libraries and the scores of the best high-scoring segment pairs from the two BLASTns are subsequently used to calculate a ratio of Insecta over Non-Insecta scores (Altschul et al., 1990). Only reads with a ratio greater than 0.8 were used in the assembly. The two libraries being non-exhaustive, we chose this threshold of 0.8 to eliminate only reads that were clearly of a non-insect origin. Selected reads were assembled with the SPAdes genome assembler using the ‘-rna’ option, allowing more heterogeneous coverage of contigs and kmer lengths of 31, 51, and 71 bases (Bankevich et al., 2012). This method successfully assembled rRNA sequences for all specimens, including a parasitic *Horreolanus* water mite (122 sequences for 28S and 114 sequences for 18S).

Initially, our filtration technique had two weaknesses. First, there is a relatively small number of complete rRNA sequences in the Insecta library from SILVA. To compensate for this, we carried out several filtration cycles, each time adding in the complete sequences produced in previous cycles to the Insecta library. Second, when our mosquito specimens were parasitized by other insects, it was not possible to bioinformatically filter out rRNA reads belonging to the parasite. For these rare cases, we used the ‘ --trusted-contigs’ option of the SPAdes assembler (Bankevich et al., 2012), giving it access to the 28S and 18S rRNA sequences of the mosquito closest in terms of taxonomic distance. By doing this, the assembler was able to reconstruct the rRNA of the mosquito as well as the rRNA of the parasitizing insect. All assembled rRNA sequences from this study have been deposited in GenBank with accession numbers OM350214–OM350327 for 18S rRNA sequences and OM542339–OM542460 for 28S rRNA sequences.

### COI amplicon sequencing

The mitochondrial COI gene was amplified from DNA samples using the universal ‘Folmer’ primer set LCO1490 (5’- GGTCAACAAATCATAAAGATATTGG -3’) and HCO2198 (5’-TAAACTTCAGGGTGACCAAAAAATCA-3’), as per standard COI marker sequencing practices, producing a 658 bp product (Folmer et al., 1994). PCRs were performed using Phusion High-Fidelity DNA Polymerase (Thermo Fisher Scientific). Every 50 µL reaction contained 10 µL of 5× High Fidelity buffer, 1 µL of 10 mM dNTPs, 2.5 µL each of 10 mM forward (LCO1490) and reverse (HCO2198) primer, 28.5 µL of water, 5 µL of DNA sample, and 0.5 µL of 2 U/µL Phusion DNA polymerase. A three-step cycling incubation protocol was used: 98°C for 30 s; 35 cycles of 98°C for 10 s, 60°C for 30 s, and 72°C for 15 s; 72°C for 5 min ending with a 4°C hold. PCR products were size-verified using gel electrophoresis and then gel-purified using the QIAquick Gel Extraction Kit (Qiagen, Hilden, Germany). Sanger sequencing of the COI amplicons were performed by Eurofins Genomics, Ebersberg, Germany.

### COI sequence analysis

Forward and reverse COI DNA sequences were end-trimmed to remove bases of poor quality (Q score <30). At the 5’ ends, sequences were trimmed at the same positions such that all forward sequences start with 5’-TTTTGG and all reverse sequences start with 5’-GGNTCT. Forward and reverse sequences were aligned using BLAST to produce a 621 bp consensus sequence. In cases where good quality sequences extends beyond 621 bp, forward and reverse sequences were assembled using Pearl (https://www.gear-genomics.com/pearl/) and manually checked for errors against trace files (Rausch et al., 2019; Rausch et al., 2020). We successfully assembled a total of 106 COI sequences. All assembled COI sequences from this study have been deposited in GenBank with accession numbers OM630610–OM630715.

### COI validation of morphology-based species identification

We analysed assembled COI sequences with BLASTn against the nucleotide collection (nr/nt) database to confirm morphology-based species identification. BLAST analyses revealed 32 cases where top hits indicated a different species identity, taking <95% nucleotide sequence similarity as the threshold to delineate distinct species (Appendix 2—table 1). In these cases, the COI sequence of the specimen was then BLAST-aligned against a GenBank record representing the morphological species to verify that the revised identity is a closer match by a significant margin, that is, more than 2% nucleotide sequence similarity. All species names reported hereafter reflect identities determined by COI sequence except for cases where COI-based identities were ambiguous, in which case morphology-based identities were retained. In cases where matches were found within a single genus but of multiple species, specimens were indicated as an unknown member of their genus (e.g., *Culex* sp.). Information of the highest-scoring references for all specimens, including details of ambiguous BLASTn results, are recorded in Appendix 2—table 1.

Within our COI sequences, we found six unidentified *Culex* species (including two that matched to GenBank entries identified only to the genus level), four unidentified *Mansonia* species, and one unidentified *Mimomyia* species. For *An. baezai*, no existing GenBank records were found at the time this analysis was performed.

### Phylogenetic analysis

Multiple sequence alignment (MSA) were performed on assembled COI and rRNA sequences using the MUSCLE software (Edgar, 2004; Madeira et al., 2019). As shown in Figure 3—figure supplement 2, the 28S rRNA sequences contain many blocks of highly conserved nucleotides, which makes the result of multiple alignment particularly evident. We therefore did not test other alignment programs. The multiple alignment of the COI amplicons is even more evident since no gaps are necessary for this alignment.

Phylogenetic tree reconstructions were performed with the MEGA X software using the maximum-likelihood method (Kumar et al., 2018). Default parameters were used with bootstrapping with 500 replications to quantify confidence level in branches. For rRNA trees, sequences belonging to an unknown species of parasitic water mite (genus *Horreolanus*) found in our specimens served as an outgroup taxon. In addition, we created and analysed a separate dataset combining our 28S rRNA sequences and full-length 28S rRNA sequences from GenBank totalling 169 sequences from 58 species (12 subgenera). To serve as outgroups for the COI tree, we included sequences obtained from GenBank of three water mite species, *Horreolanus orphanus* (KM101004), *Sperchon fuxiensis* (MH916807), and *Arrenurus* sp. (MN362807).

## Data Availability

Multiple sequence alignment files are included as source data files. All sequences generated in this study have been deposited in GenBank under the accession numbers OM350214–OM350327 for 18S rRNA sequences, OM542339–OM542460 for 28S rRNA sequences, and OM630610–OM630715 for COI sequences.

## Additional files

Transparent reporting form

## Appendix 1

**Appendix 1—table 1.** Taxonomic and sampling information on mosquito specimens and associated accession numbers of their cytochrome *c* oxidase I (COI), 18S rRNA, and 28S rRNA sequences (XLSX).

Sequence ID	Taxonomy [Genus (subgenus) species]	Origin	Collection site	Collection period	Blood engorged (Y/N)	Sample ID	COI accession number	18S rRNA accession number	28S rRNA accession number
Ae_albopictus_KH_S1	*Aedes* (*Stegomyia*) *albopictus*	Cambodia	Rattanakiri	Dec 2019	N	1	OM630613	OM350214	OM542460
Ae_albopictus_KH_S2	*Aedes* (*Stegomyia*) *albopictus*	Cambodia	Rattanakiri	Dec 2019	N	2	OM630614	OM350220	OM542373
Ae_albopictus_KH_S3	*Aedes* (*Stegomyia*) *albopictus*	Cambodia	Rattanakiri	Dec 2019	N	3	OM630615	OM350316	OM542374
An_baezai_KH_S4	*Anopheles* (*Anopheles*) *baezai*	Cambodia	Koh Kong	Mar 2019	N	4	OM630631	OM350327	OM542357
An_baezai_KH_S5	*Anopheles* (*Anopheles*) *baezai*	Cambodia	Koh Kong	Mar 2019	N	5	OM630632	OM350233	OM542440
An_baezai_KH_S6	*Anopheles* (*Anopheles*) *baezai*	Cambodia	Koh Kong	Mar 2019	N	6	OM630633	OM350234	OM542358
Cx_pseudovishnui_KH_S7	*Culex* (*Culex*) *pseudovishnui*	Cambodia	Rattanakiri	Dec 2019	N	7	OM630689	OM350285	OM542413
Cx_pseudovishnui_KH_S8	*Culex* (*Culex*) *pseudovishnui*	Cambodia	Rattanakiri	Dec 2019	N	8	OM630690	OM350286	OM542414
Cx_pseudovishnui_KH_S9	*Culex* (*Culex*) *pseudovishnui*	Cambodia	Rattanakiri	Dec 2019	N	9	OM630691	OM350287	OM542415
Ma_indiana_KH_S10	*Mansonia* (*Mansonioides*) *indiana*	Cambodia	Battambong	Nov 2019	N	10	OM630698	OM350295	OM542422
Ma_uniformis_KH_S11	*Mansonia* (*Mansonioides*) *uniformis*	Cambodia	Battambong	Nov 2019	N	11	OM630699	OM350296	OM542423
Ma_indiana_KH_S12	*Mansonia* (*Mansonioides*) *indiana*	Cambodia	Battambong	Nov 2019	N	12	OM630700	OM350297	OM542424
Cx_sp.1_KH_S13	*Culex* sp.1	Cambodia	Prek Toal	Feb 2019	N	13	OM630672	OM350267	OM542395
Cx_orientalis_KH_S14	*Culex* (*Culex*) *orientalis*	Cambodia	Prek Toal	Feb 2019	N	14	OM630673	OM350268	OM542396
Ma_uniformis_KH_S15	*Mansonia* (*Mansonioides*) *uniformis*	Cambodia	Battambong	Nov 2019	N	15	OM630705	OM350303	OM542430
Ma_uniformis_KH_S16	*Mansonia* (*Mansonioides*) *uniformis*	Cambodia	Battambong	Nov 2019	N	16	OM630706	OM350305	OM542432
Ma_uniformis_KH_S17	*Mansonia* (*Mansonioides*) *uniformis*	Cambodia	Battambong	Nov 2019	N	17	OM630707	OM350304	OM542431
Cx_bitaeniorhynchus_KH_S18	*Culex* (*Oculeomyia*) *bitaeniorhynchus*	Cambodia	Battambong	Nov 2019	N	18	OM630656	OM350255	OM542381
Cx_bitaeniorhynchus_KH_S19	*Culex* (*Oculeomyia*) *bitaeniorhynchus*	Cambodia	Battambong	Nov 2019	N	19	OM630657	OM350256	OM542382
Cx_bitaeniorhynchus_KH_S20	*Culex* (*Oculeomyia*) *bitaeniorhynchus*	Cambodia	Battambong	Nov 2019	N	20	OM630658	OM350257	OM542383, OM542384
Cx_tritaeniorhynchus_KH_S21	*Culex* (*Culex*) *tritaeniorhynchus*	Cambodia	Battambong	Nov 2019	N	21	OM630680	OM350277	OM542404
Cx_tritaeniorhynchus_KH_S22	*Culex* (*Culex*) *tritaeniorhynchus*	Cambodia	Battambong	Nov 2019	N	22	OM630681	OM350278	OM542405
Cx_tritaeniorhynchus_KH_S23	*Culex* (*Culex*) *tritaeniorhynchus*	Cambodia	Battambong	Nov 2019	N	23	OM630682	OM350279	OM542406
Ae_aegypti_CF_S24	*Aedes* (*Stegomyia*) *aegypti*	Central African Republic	Pissa	Jun 2019	N	24	OM630610	OM350314	OM542339
Ae_aegypti_CF_S25	*Aedes* (*Stegomyia*) *aegypti*	Central African Republic	Pissa	Jun 2019	N	25	OM630611	OM350215	OM542340
Ae_aegypti_CF_S26	*Aedes* (*Stegomyia*) *aegypti*	Central African Republic	Pissa	Jun 2019	N	26	OM630612	OM350216	OM542341
Ae_simpsoni_CF_S27	*Aedes* (*Stegomyia*) *simpsoni*	Central African Republic	Pissa	Jun 2019	N	27	OM630619	OM350221	OM542345
Ae_simpsoni_CF_S28	*Aedes* (*Stegomyia*) *simpsoni*	Central African Republic	Pissa	Jun 2019	N	28	OM630620	OM350222	OM542346
Ae_simpsoni_CF_S29	*Aedes* (*Stegomyia*) *simpsoni*	Central African Republic	Pissa	Jun 2019	N	29	OM630621	OM350223	OM542347
Ae_vittatus_CF_S30	*Aedes* (*Fredwardsius*) *vittatus*	Central African Republic	Gbozo	Aug 2019	Y	30	OM630628	OM350230	OM542439
Ae_vittatus_CF_S31	*Aedes* (*Fredwardsius*) *vittatus*	Central African Republic	Gbozo	Aug 2019	N	31	OM630629	OM350231	OM542355
Ae_vittatus_CF_S32	*Aedes* (*Fredwardsius*) *vittatus*	Central African Republic	Gbozo	Aug 2019	N	32	OM630630	OM350232	OM542356
Ma_sp.1_CF_S33	*Mansonia* sp.1	Central African Republic	Bayanga	Nov 2019	Y	33	N/A	OM350294	OM542449
Ma_sp.2_CF_S34	*Mansonia* sp.2	Central African Republic	Bayanga	Nov 2019	Y	34	N/A	OM350322	OM542450, OM542456
Ho_sp.1_CF_S34	*Horreolanus* sp.1	Central African Republic	Bayanga	Nov 2019	–	34	N/A	OM350325	OM542457
Ho_sp.2_CF_S34	*Horreolanus* sp.2	Central African Republic	Bayanga	Nov 2019	–	34	N/A	OM350326	OM542458
Ma_sp.3_CF_S35	*Mansonia* sp.3	Central African Republic	Bayanga	Nov 2019	Y	35	N/A	OM350323	OM542451
Ae_albopictus_CF_S36	*Aedes* (*Stegomyia*) *albopictus*	Central African Republic	Pissa	Jun 2019	N	36	OM630616	OM350217	OM542342
Ae_albopictus_CF_S37	*Aedes* (*Stegomyia*) *albopictus*	Central African Republic	Pissa	Jun 2019	N	37	OM630617	OM350218	OM542343
Ae_albopictus_CF_S38	*Aedes* (*Stegomyia*) *albopictus*	Central African Republic	Pissa	Jun 2019	N	38	OM630618	OM350219	OM542344
An_coustani_CF_S39	*Anopheles* (*Anopheles*) *coustani*	Central African Republic	Pissa	Jan 2020	N	39	OM630634	OM350235	OM542359
An_coustani_CF_S40	*Anopheles* (*Anopheles*) *coustani*	Central African Republic	Pissa	Jan 2020	N	40	OM630635	OM350236	OM542360
An_coustani_CF_S41	*Anopheles* (*Anopheles*) *coustani*	Central African Republic	Pissa	Jan 2020	N	41	OM630636	OM350237	OM542361
An_funestus_CF_S42	*Anopheles* (*Cellia*) *funestus*	Central African Republic	Pissa	Jun 2019	Y	42	OM630640	OM350241	OM542365
An_funestus_CF_S43	*Anopheles* (*Cellia*) *funestus*	Central African Republic	Pissa	Jun 2019	Y	43	OM630641	OM350242	OM542366
An_funestus_CF_S44	*Anopheles* (*Cellia*) *funestus*	Central African Republic	Pissa	Jun 2019	Y	44	OM630642	OM350243	OM542367
An_gambiae_CF_S45	*Anopheles* (*Cellia*) *gambiae*	Central African Republic	Pissa	Jun 2019	Y	45	OM630645	OM350245	OM542369, OM542370
An_gambiae_CF_S46	*Anopheles* (*Cellia*) *gambiae*	Central African Republic	Pissa	Jun 2019	Y	46	OM630646	OM350246	OM542371
An_gambiae_CF_S47	*Anopheles* (*Cellia*) *gambiae*	Central African Republic	Pissa	Jun 2019	Y	47	N/A	OM350247	OM542372
Cx_sp.2_CF_S48	*Culex* sp.2	Central African Republic	Bayanga	Nov 2019	Y	48	OM630669	OM350269	OM542446
Cx_sp.2_CF_S49	*Culex* sp.2	Central African Republic	Bayanga	Nov 2019	Y	49	OM630670	OM350315	OM542397
Cx_sp.2_CF_S50	*Culex* sp.2	Central African Republic	Bayanga	Nov 2019	Y	50	OM630671	OM350270	OM542398
Ma_uniformis_CF_S51	*Mansonia* (*Mansonioides*) *uniformis*	Central African Republic	Bouar	May 2019	Y	51	N/A	OM350301	OM542428
Cx_duttoni_CF_S52	*Culex* (*Culex*) *duttoni*	Central African Republic	Mbaiki	Jan 2019	Y	52	OM630704	OM350302	OM542429
Er_intermedius_CF_S54	*Eretmapodites intermedius*	Central African Republic	Pissa	Jun 2019	N	54	OM630692	OM350288	OM542416
Er_intermedius_CF_S55	*Eretmapodites intermedius*	Central African Republic	Pissa	Jun 2019	N	55	OM630693	OM350289	OM542417
Er_intermedius_CF_S56	*Eretmapodites intermedius*	Central African Republic	Pissa	Jun 2019	N	56	OM630694	OM350290	OM542418
Cx_antennatus_MG_S57	*Culex* (*Culex*) *antennatus*	Madagascar	Ambato Boeny	Feb 2019	N	57	OM630653	OM350253	OM542379
Cx_antennatus_MG_S58	*Culex* (*Culex*) *antennatus*	Madagascar	Ambato Boeny	Feb 2019	N	58	OM630654	OM350319	OM542444
Cx_antennatus_MG_S59	*Culex* (*Culex*) *antennatus*	Madagascar	Ambato Boeny	Feb 2019	N	59	OM630655	OM350254	OM542380
Cx_perexiguus_MG_S60	*Culex* (*Culex*) *perexiguus*	Madagascar	Amparafaravola	Feb 2019	N	60	OM630660	OM350258	OM542386
Cx_sp.3_MG_S61	*Culex* sp.3	Madagascar	Ambato Boeny	Aug 2019	N	61	OM630661	OM350259	OM542387
Cx_sp.4_MG_S62	*Culex* sp.4	Madagascar	Ambato Boeny	Aug 2019	N	62	OM630662	OM350260	OM542388
Cx_sp.3_MG_S63	*Culex* sp.3	Madagascar	Ambato Boeny	Feb 2019	N	63	OM630686	OM350282	OM542410
Mi_sp.1_MG_S64	*Mimomyia* sp.1	Madagascar	Ambato Boeny	Feb 2019	N	64	OM630687	OM350283	OM542411
Cx_sp.3_MG_S65	*Culex* sp.3	Madagascar	Ambato Boeny	Feb 2019	N	65	OM630688	OM350284	OM542412
An_coustani_MG_S66	*Anopheles* (*Anopheles*) *coustani*	Madagascar	Ambato Boeny	Feb 2019	N	66	OM630637	OM350238	OM542362
An_coustani_MG_S67	*Anopheles* (*Anopheles*) *coustani*	Madagascar	Ambato Boeny	Feb 2019	N	67	OM630638	OM350239	OM542363
An_squamosus_MG_S68	*Anopheles* (*Cellia*) *squamosus*	Madagascar	Ambato Boeny	Feb 2019	N	68	OM630639	OM350240	OM542364
An_funestus_MG_S69	*Anopheles* (*Cellia*) *funestus*	Madagascar	Ambato Boeny	Feb 2019	N	69	OM630643	OM350244	OM542368
An_funestus_MG_S70	*Anopheles* (*Cellia*) *funestus*	Madagascar	Ambato Boeny	Feb 2020	N	70	OM630644	OM350317	OM542441
An_gambiae_MG_S71	*Anopheles* (*Cellia*) *gambiae*	Madagascar	Ambato Boeny	Feb 2019	N	71	OM630647	OM350249	OM542442
An_gambiae_MG_S72	*Anopheles* (*Cellia*) *gambiae*	Madagascar	Ambato Boeny	Feb 2019	N	72	OM630648	OM350248	OM542443
An_gambiae_MG_S73	*Anopheles* (*Cellia*) *gambiae*	Madagascar	Ambato Boeny	Feb 2019	N	73	OM630649	OM350318	OM542459
Cx_quinquefasciatus_MG_S74	*Culex* (*Culex*) *quinquefasciatus*	Madagascar	Amparafaravola	Feb 2019	N	74	OM630674	OM350271	OM542399
Cx_quinquefasciatus_MG_S75	*Culex* (*Culex*) *quinquefasciatus*	Madagascar	Amparafaravola	Feb 2019	N	75	OM630675	OM350272	OM542447
Cx_perexiguus_MG_S76	*Culex* (*Culex*) *perexiguus*	Madagascar	Mampikony	Aug 2019	N	76	OM630676	OM350273	OM542400
Ma_uniformis_MG_S77	*Mansonia* (*Mansonioides*) *uniformis*	Madagascar	Ambato Boeny	Feb 2019	N	77	OM630708	OM350306	OM542433
Ma_uniformis_MG_S78	*Mansonia* (*Mansonioides*) *uniformis*	Madagascar	Ambato Boeny	Feb 2019	N	78	OM630709	OM350307	OM542434
Ma_uniformis_MG_S79	*Mansonia* (*Mansonioides*) *uniformis*	Madagascar	Ambato Boeny	Feb 2019	N	79	OM630710	OM350308	OM542435
Cx_poicilipes_MG_S82	*Culex poicilipes*	Madagascar	Mampikony	Feb 2019	N	82	OM630659	OM350320	OM542385, OM542445
Mi_mediolineata_MG_S83	*Mimomyia mediolineata*	Madagascar	Ambato Boeny	Feb 2019	N	83	OM630683	OM350280	OM542407
Cx_neavei_MG_S84	*Culex* (*Culex*) *neavei*	Madagascar	Ambato Boeny	Feb 2019	N	84	OM630684	OM350281	OM542408, OM542409
Ae_scapularis_GF_S85	*Aedes* (*Ochlerotatus*) *scapularis*	French Guiana	Hameau Prefontaine	Jul 2019	N	85	OM630624	OM350224	OM542348, OM542349
Ae_scapularis_GF_S86	*Aedes* (*Ochlerotatus*) *scapularis*	French Guiana	Hameau Prefontaine	Jul 2019	N	86	OM630622	OM350225	OM542350
Ae_scapularis_GF_S87	*Aedes* (*Ochlerotatus*) *scapularis*	French Guiana	Hameau Prefontaine	Jul 2019	N	87	OM630623	OM350226	OM542351
Ae_serratus_GF_S88	*Aedes* (*Ochlerotatus*) *serratus*	French Guiana	Hameau Prefontaine	Nov 2020	N	88	OM630625	OM350227	OM542352
Ae_serratus_GF_S89	*Aedes* (*Ochlerotatus*) *serratus*	French Guiana	Hameau Prefontaine	Nov 2020	N	89	OM630626	OM350228	OM542353
Ae_serratus_GF_S90	*Aedes* (*Ochlerotatus*) *serratus*	French Guiana	Hameau Prefontaine	Nov 2020	N	90	OM630627	OM350229	OM542354
Cq_venezuelensis_GF_S91	*Coquillettidia venezuelensis*	French Guiana	Hameau Prefontaine	Jul 2019	N	91	OM630650	OM350250	OM542375
Cq_venezuelensis_GF_S92	*Coquillettidia venezuelensis*	French Guiana	Hameau Prefontaine	Jul 2019	N	92	OM630651	OM350251	OM542376
Cq_venezuelensis_GF_S93	*Coquillettidia venezuelensis*	French Guiana	Hameau Prefontaine	Jul 2019	N	93	OM630652	OM350252	OM542377, OM542378
Cx_portesi_GF_S94	*Culex* sp. BTLHVDV-2014	French Guiana	Hameau Prefontaine	Jul 2019	N	94	OM630666	OM350264	OM542392
Cx_portesi_GF_S95	*Culex* sp. BTLHVDV-2014	French Guiana	Hameau Prefontaine	Jul 2019	N	95	OM630667	OM350265	OM542393
Cx_portesi_GF_S96	*Culex* sp. BTLHVDV-2014	French Guiana	Hameau Prefontaine	Jul 2019	N	96	OM630668	OM350266	OM542394
Cx_spissipes_GF_S97	*Culex* (*Melanoconion*) sp. DJS-2020	French Guiana	Hameau Prefontaine	Jul 2019	N	97	OM630677	OM350274	OM542401
Cx_spissipes_GF_S98	*Culex* (*Melanoconion*) sp. DJS-2020	French Guiana	Hameau Prefontaine	Jul 2019	N	98	OM630678	OM350275	OM542402
Cx_spissipes_GF_S99	*Culex* (*Melanoconion*) sp. DJS-2020	French Guiana	Hameau Prefontaine	Jul 2019	N	99	OM630679	OM350276	OM542403
Li_durhamii_GF_S100	*Limatus durhamii*	French Guiana	Hameau Prefontaine	Jul 2019	N	100	OM630695	OM350291	OM542419
Li_durhamii_GF_S101	*Limatus durhamii*	French Guiana	Hameau Prefontaine	Jul 2019	N	101	OM630696	OM350292	OM542420
Li_durhamii_GF_S102	*Limatus durhamii*	French Guiana	Hameau Prefontaine	Jul 2019	N	102	OM630697	OM350293	OM542421
Ma_sp.4_GF_S103	*Mansonia* sp.4	French Guiana	Hameau Prefontaine	Jan 2020	N	103	OM630701	OM350298	OM542425
Ma_sp.4_GF_S104	*Mansonia* sp.4	French Guiana	Hameau Prefontaine	Jan 2020	N	104	OM630702	OM350299	OM542426
Ma_titillans_GF_S105	*Mansonia* (*Mansonia*) *titillans*	French Guiana	Hameau Prefontaine	Jan 2020	N	105	OM630703	OM350300	OM542427
Cx_pedroi_GF_S106	*Culex* (*Melanoconion*) *pedroi*	French Guiana	Hameau Prefontaine	Nov 2020	N	106	OM630663	OM350261	OM542389
Cx_pedroi_GF_S107	*Culex* (*Melanoconion*) *pedroi*	French Guiana	Hameau Prefontaine	Nov 2020	N	107	OM630664	OM350262	OM542390
Cx_pedroi_GF_S108	*Culex* (*Melanoconion*) *pedroi*	French Guiana	Hameau Prefontaine	Nov 2020	N	108	OM630665	OM350263	OM542391
Ps_ferox_GF_S109	*Psorophora ferox*	French Guiana	Iracoubo	2009	N	109	OM630711	OM350309	OM542436
Ps_ferox_GF_S110	*Psorophora ferox*	French Guiana	Iracoubo	2009	N	110	OM630712	OM350310	OM542437
Ps_ferox_GF_S111	*Psorophora ferox*	French Guiana	Iracoubo	2009	N	111	OM630713	OM350324	OM542452
Ur_geometrica_GF_S112	*Uranotaenia* (*Uranotaenia*) *geometrica*	French Guiana		2010	N	112	OM630714	OM350311	OM542453
Ur_geometrica_GF_S113	*Uranotaenia* (*Uranotaenia*) *geometrica*	French Guiana		2010	N	113	N/A	OM350312	OM542454
Ur_geometrica_GF_S114	*Uranotaenia* (*Uranotaenia*) *geometrica*	French Guiana		2010	N	114	OM630715	OM350313	OM542438, OM542455
Cx_tritaeniorhynchus_MG_S115	*Culex* (*Culex*) *tritaeniorhynchus*	Madagascar	Ambato Boeny	Feb 2019	N	115	OM630685	OM350321	OM542448

## Appendix 2

**Appendix 2—table 1.** Cytochrome *c* oxidase I (COI) sequence BLAST analyses summary (XLSX).

Sequence ID	Sequence length	Morphological identification	BLASTn top hit species	BLASTn top hit accession	Query coverage	% identity	Comments
Ae_albopictus_KH_S1	699	*Aedes albopictus*	*Aedes albopictus*	MK714006.1	99%	99.71%	
Ae_albopictus_KH_S2	695	*Aedes albopictus*	*Aedes albopictus*	MK714006.1	100%	99.71%	
Ae_albopictus_KH_S3	695	*Aedes albopictus*	*Aedes albopictus*	MK714006.1	100%	99.71%	
An_baezai_KH_S4	658	*Anopheles baezai*	*Anopheles darlingi*	MF381626.1	100%	92.71%	*Anopheles baezai* not found in GenBank
An_baezai_KH_S5	670	*Anopheles baezai*	*Anopheles darlingi*	MF381626.1	99%	92.81%	*Anopheles baezai* not found in GenBank
An_baezai_KH_S6	659	*Anopheles baezai*	*Anopheles darlingi*	MF381626.1	100%	92.72%	*Anopheles baezai* not found in GenBank
Cx_pseudovishnui_KH_S7	660	*Culex vishnui*	*Culex pseudovishnui*	MW321882.1	98%	98.92%	95% similarity to *Culex vishnui*, 94% similarity to *Culex tritaeniorhynchus*
Cx_pseudovishnui_KH_S8	659	*Culex vishnui*	*Culex pseudovishnui*	MW321882.1	98%	99.38%	95% similarity to *Culex vishnui*, 94% similarity to *Culex tritaeniorhynchus*
Cx_pseudovishnui_KH_S9	659	*Culex vishnui*	*Culex pseudovishnui*	MW321882.1	98%	98.92%	95% similarity to *Culex vishnui*, 94% similarity to *Culex tritaeniorhynchus*
Ma_indiana_KH_S10	660	*Mansonia indiana*	*Mansonia indiana*	MK637632.1	98.00%	99.54%	
Ma_uniformis_KH_S11	686	*Mansonia indiana*	*Mansonia uniformis*	MK757484.1	99%	99.71%	89.99% similarity to *Mansonia indiana* MK637632.1
Ma_indiana_KH_S12	693	*Mansonia indiana*	*Mansonia indiana*	MK637632.1	97%	99.41%	
Cx_sp.1_KH_S13	687	*Culex quinquefasciatus*	*Culex* (*Lophoceraomyia*) sp.5 HY-2020	MW321904.1	98%	94.39%	90% similarity to *Culex quinquefasciatus* GU188856.2
Cx_orientalis_KH_S14	662	*Culex quinquefasciatus*	*Culex orientalis*	MW228488.1	97%	98.29%	
Ma_uniformis_KH_S15	658	*Mansonia uniformis*	*Mansonia uniformis*	MK757484.1	100.00%	99.54%	
Ma_uniformis_KH_S16	654	*Mansonia uniformis*	*Mansonia uniformis*	MK757484.1	100.00%	99.39%	
Ma_uniformis_KH_S17	657	*Mansonia uniformis*	*Mansonia uniformis*	MK757484.1	99.00%	99.54%	
Cx_bitaeniorhynchus_KH_S18	658	*Culex bitaeniorhynchus*	*Culex bitaeniorhynchus*	HQ398898.1	97.00%	99.69%	
Cx_bitaeniorhynchus_KH_S19	650	*Culex bitaeniorhynchus*	*Culex bitaeniorhynchus*	HQ398898.1	98.00%	99.84%	
Cx_bitaeniorhynchus_KH_S20	652	*Culex bitaeniorhynchus*	*Culex bitaeniorhynchus*	HQ398898.1	98.00%	99.38%	
Cx_tritaeniorhynchus_KH_S21	695	*Culex tritaeniorhynchus*	*Culex vishnui* or *Culex tritaeniorhynchus*	MH374857.1	100%	99.57%	99.69% similarity to *Culex tritaeniorhynchus* MF179213.1
Cx_tritaeniorhynchus_KH_S22	690	*Culex tritaeniorhynchus*	*Culex vishnui* or *Culex tritaeniorhynchus*	MT876103.1	100%	99.57%	
Cx_tritaeniorhynchus_KH_S23	663	*Culex tritaeniorhynchus*	*Culex tritaeniorhynchus*	MT876103.1	99%	98.79%	
Ae_aegypti_CF_S24	689	*Aedes aegypti*	*Aedes aegypti*	MN299016.1	100%	99.56%	
Ae_aegypti_CF_S25	660	*Aedes aegypti*	*Aedes aegypti*	MN299024.1	100.00%	99.70%	
Ae_aegypti_CF_S26	660	*Aedes aegypti*	*Aedes aegypti*	MN299024.1	100.00%	99.70%	
Ae_simpsoni_CF_S27	644	*Aedes opok*	*Aedes simpsoni*	LC473669.1	97.00%	97.77%	*Aedes opok* not found in GenBank, sequence has 90% and 89% similarity to *Aedes luteocephalus* and *Aedes africanus*, sister species of *Aedes opok*.
Ae_simpsoni_CF_S28	649	*Aedes opok*	*Aedes simpsoni*	MN552302.1	99.00%	100.00%	*Aedes*. *opok* not found in GenBank, sequence has 90% and 89% similarity to *Aedes luteocephalus* and *Aedes africanus*, sister species of *Aedes opok*.
Ae_simpsoni_CF_S29	627	*Aedes opok*	*Aedes simpsoni*	MN552302.1	98.00%	98.87%	*Aedes opok* not found in GenBank, sequence has 90% and 89% similarity to *Aedes luteocephalus* and *Aedes africanus*, sister species of *Aedes opok*.
Ae_vittatus_CF_S30	623	*Aedes vittatus*	*Aedes vittatus*	MN552298.1	100.00%	99.84%	
Ae_vittatus_CF_S31	622	*Aedes vittatus*	*Aedes vittatus*	MN552298.1	100.00%	99.68%	
Ae_vittatus_CF_S32	621	*Aedes vittatus*	*Aedes vittatus*	MN552298.1	100.00%	99.68%	
Ma_sp.1_CF_S33	–	*Mansonia africana*	–	–	–	–	No COI obtained
Ma_sp.2_CF_S34	–	*Mansonia africana*	–	–	–	–	No COI obtained
Ma_sp.3_CF_S35	–	*Mansonia africana*	–	–	–	–	No COI obtained
Ae_albopictus_CF_S36	627	*Aedes albopictus*	*Aedes albopictus*	MK995332.1	100.00%	99.84%	
Ae_albopictus_CF_S37	621	*Aedes albopictus*	*Aedes albopictus*	MK995332.1	100.00%	100.00%	
Ae_albopictus_CF_S38	621	*Aedes albopictus*	*Aedes albopictus*	MK995332.1	100.00%	100.00%	
An_coustani_CF_S39	621	*Anopheles coustani*	*Anopheles coustani*	MK585968.1	100.00%	99.84%	
An_coustani_CF_S40	621	*Anopheles coustani*	*Anopheles coustani*	MK585959.1	100.00%	99.03%	
An_coustani_CF_S41	699	*Anopheles coustani*	*Anopheles coustani*	MK585968.1	94.00%	99.70%	
An_funestus_CF_S42	696	*Anopheles funestus*	*Anopheles funestus*	MK300231.1	100.00%	99.71%	
An_funestus_CF_S43	660	*Anopheles funestus*	*Anopheles funestus*	MT375215.1	100.00%	99.85%	
An_funestus_CF_S44	658	*Anopheles funestus*	*Anopheles funestus*	MT375215.1	100.00%	99.70%	
An_gambiae_CF_S45	660	*Anopheles gambiae*	*Anopheles gambiae*	MG930895.1	86.00%	99.79%	
An_gambiae_CF_S46	659	*Anopheles gambiae*	*Anopheles gambiae*	MT375223.1	89.00%	100.00%	
An_gambiae_CF_S47	–	*Anopheles gambiae*	–	–	–	–	No COI obtained
Cx_sp.2_CF_S48	653	*Culex quinquefasciatus*	*Culex corniger*	KM593015.1	100.00%	94.95%	94% similarity to all other *Culex* species
Cx_sp.2_CF_S49	660	*Culex quinquefasciatus*	*Culex nigripalpus*	KM593058.1	99.00%	94.65%	94% similarity to all other *Culex* species
Cx_sp.2_CF_S50	658	*Culex quinquefasciatus*	*Culex bidens*	MH931446.1	100.00%	94.68%	94% similarity to all other *Culex* species
Ma_uniformis_CF_S51	–	*Mansonia uniformis*	–	–	–	–	No COI obtained
Cx_duttoni_CF_S52	621	*Mansonia uniformis*	*Culex duttoni*	LC473629.1	100.00%	99.68%	
Er_intermedius_CF_S54	620	*Eretmapodites* sp.	*Eretmapodites intermedius*	MN552305.1	100.00%	99.52%	
Er_intermedius_CF_S55	621	*Eretmapodites* sp.	*Eretmapodites intermedius*	MN552305.1	100.00%	99.68%	
Er_intermedius_CF_S56	621	*Eretmapodites* sp.	*Eretmapodites intermedius*	MN552305.1	100.00%	99.68%	
Cx_antennatus_MG_S57	621	*Culex antennatus*	*Culex antennatus*	LC473659.1	100.00%	100.00%	
Cx_antennatus_MG_S58	621	*Culex antennatus*	*Culex antennatus*	LC473659.1	100.00%	100.00%	
Cx_antennatus_MG_S59	621	Culex. antennatus	*Culex antennatus*	LC473659.1	100.00%	100.00%	
Cx_perexiguus_MG_S60	621	*Culex decens*	*Culex perexiguus*	LC473634.1	100.00%	99.84%	
Cx_sp.3_MG_S61	685	*Culex decens*	Unknown *Culex* species	KU380436.1	96.00%	96.05%	
Cx_sp.4_MG_S62	687	*Culex decens*	Unknown *Culex* species	MT993494.1	99.00%	95.63%	
Cx_sp.3_MG_S63	687	*Culex univittatus*	Unknown *Culex* species	KU380436.1	95.00%	96.50%	
Mi_sp.1_MG_S64	694	*Culex univittatus*	*Mimomyia mimomyiaformis*	LC473719.1	94.00%	92.55%	Unknown *Mimomyia* species
Cx_sp.3_MG_S65	691	*Culex univittatus*	Unknown *Culex* species	KU380436.1	95.00%	96.66%	
An_coustani_MG_S66	669	*Anopheles coustani*	*Anopheles coustani*	NC_050693.1	99.00%	99.40%	
An_coustani_MG_S67	659	*Anopheles coustani*	*Anopheles coustani*	NC_050693.1	99.00%	99.08%	
An_squamosus_MG_S68	653	*Anopheles coustani*	*Anopheles squamosus*	MK776741.1	100.00%	100.00%	
An_funestus_MG_S69	654	*Anopheles funestus*	*Anopheles funestus*	MT375215.1	100.00%	99.85%	
An_funestus_MG_S70	654	*Anopheles funestus*	*Anopheles funestus*	MG742199.1	100.00%	99.69%	
An_gambiae_MG_S71	654	*Anopheles gambiae*	*Anopheles gambiae*	MT375222.1	100.00%	99.85%	
An_gambiae_MG_S72	654	*Anopheles gambiae*	*Anopheles gambiae*	MT375222.1	100.00%	99.85%	
An_gambiae_MG_S73	622	*Anopheles gambiae*	*Anopheles gambiae*	MT375222.1	100.00%	100.00%	
Cx_quinquefasciatus_MG_S74	654	*Culex quinquefasciatus*	*Culex pipiens*	MT199095.1	100.00%	100.00%	99.85% similarity to *Culex quinquefasciatus*
Cx_quinquefasciatus_MG_S75	647	*Culex quinquefasciatus*	*Culex quinquefasciatus*	MH423504.1	100.00%	98.15%	Also 98% similarity to *Culex pipiens*
Cx_perexiguus_MG_S76	621	*Culex quinquefasciatus*	*Culex perexiguus*	LC473634.1	100.00%	99.52%	Same SNPs to *Culex pipiens* MH374861.1
Ma_uniformis_MG_S77	621	*Mansonia uniformis*	*Mansonia uniformis*	KU187165.1	100.00%	100.00%	
Ma_uniformis_MG_S78	621	*Mansonia uniformis*	*Mansonia uniformis*	KU187165.1	100.00%	100.00%	
Ma_uniformis_MG_S79	626	*Mansonia uniformis*	*Mansonia uniformis*	KU187157.1	100.00%	99.68%	
Cx_poicilipes_MG_S82	689	*Culex bitaeniorhynchus*	*Culex poicilipes*	LC473618.1	95.00%	99.70%	
Mi_mediolineata_MG_S83	694	*Culex tritaeniorhynchus*	*Mimomyia mediolineata*	LC473723.1	94.00%	99.39%	
Cx_neavei_MG_S84	671	*Culex tritaeniorhynchus*	*Culex neavei*	LC473635.1	98.00%	99.85%	
Ae_scapularis_GF_S85	659	*Aedes scapularis*	*Aedes scapularis*	MN997484.1	97.00%	98.76%	
Ae_scapularis_GF_S86	658	*Aedes scapularis*	*Aedes scapularis*	MF172265.1	97.00%	99.38%	
Ae_scapularis_GF_S87	654	*Aedes scapularis*	*Aedes scapularis*	MF172265.1	98.00%	99.22%	
Ae_serratus_GF_S88	660	*Aedes serratus*	*Aedes serratus*	MF172269.1	97.00%	98.91%	
Ae_serratus_GF_S89	660	*Aedes serratus*	*Aedes serratus*	MF172268.1	97.00%	99.22%	
Ae_serratus_GF_S90	654	*Aedes serratus*	*Aedes serratus*	MF172268.1	98.00%	99.07%	
Cq_venezuelensis_GF_S91	658	*Coquillettidia venezuelensis*	*Coquillettidia venezuelensis*	MN997703.1	97.00%	97.98%	
Cq_venezuelensis_GF_S92	621	*Coquillettidia venezuelensis*	*Coquillettidia. venezuelensis*	MN997703.1	100.00%	98.07%	
Cq_venezuelensis_GF_S93	621	*Coquillettidia venezuelensis*	*Coquillettidia venezuelensis*	MN997703.1	100.00%	97.75%	
Cx_portesi_GF_S94	653	*Culex portesi*	*Culex portesi*	in-house reference library		98.5–100%	Reference sequence provided by Amandine Guidez, IP Guyane
Cx_portesi_GF_S95	693	*Culex portesi*	*Culex portesi*	in-house reference library		98.5–100%	Reference sequence provided by Amandine Guidez, IP Guyane
Cx_portesi_GF_S96	687	*Culex portesi*	*Culex portesi*	in-house reference library		98.5–100%	Reference sequence provided by Amandine Guidez, IP Guyane
Cx_spissipes_GF_S97	672	*Culex spissipes*	*Culex spissipes*	in-house reference library		98.5–100%	Reference sequence provided by Amandine Guidez, IP Guyane
Cx_spissipes_GF_S98	663	*Culex spissipes*	*Culex spissipes*	in-house reference library		98.5–100%	Reference sequence provided by Amandine Guidez, IP Guyane
Cx_spissipes_GF_S99	660	*Culex spissipes*	*Culex spissipes*	in-house reference library		98.5–100%	Reference sequence provided by Amandine Guidez, IP Guyane
Li_durhamii_GF_S100	653	*Lmatus durhamii*	*Limatus durhamii*	MF172330.1	98.00%	99.84%	
Li_durhamii_GF_S101	621	*Limatus durhamii*	*Limatus durhamii*	MF172330.1	100.00%	100.00%	
Li_durhamii_GF_S102	699	*Limatus durhamii*	*Limatus durhamii*	MF172330.1	94.00%	100.00%	
Ma_sp.4_GF_S103	621	*Mansonia titillans*	*Mansonia* sp.	MT329066.1	100.00%	99.84%	87.12% similarity to *Mansonia titillans* MN968244.1
Ma_sp.4_GF_S104	695	*Mansonia titillans*	*Mansonia* sp.	MT329066.1	95.00%	99.85%	87.39% to *Mansonia titillans* MN968244.1
Ma_titillans_GF_S105	669	*Mansonia titillans*	*Mansonia titillans*	MN968244.1	98.00%	99.70%	
Cx_pedroi_GF_S106	653	*Culex pedroi*	*Culex pedroi*	KX779887.1	98.00%	98.60%	
Cx_pedroi_GF_S107	661	*Culex pedroi*	*Culex pedroi*	KX779887.1	97.00%	98.76%	
Cx_pedroi_GF_S108	621	*Culex pedroi*	*Culex pedroi*	KX779887.1	99.00%	98.87%	
Ps_ferox_GF_S109	633	*Psorophora ferox*	*Psorophora ferox*	MF172349.1	100.00%	99.68%	
Ps_ferox_GF_S110	621	*Psorophora ferox*	*Psorophora ferox*	MF172349.1	100.00%	99.68%	
Ps_ferox_GF_S111	621	*Psorophora ferox*	*Psorophora ferox*	MF172347.1	99.00%	99.51%	
Ur_geometrica_GF_S112	621	*Uranotaenia geometrica*	*Uranotaenia geometrica*	NC_044662.1	100.00%	100.00%	
Ur_geometrica_GF_S113	–	*Uranotaenia geometrica*	–	–	–	–	No COI obtained
Ur_geometrica_GF_S114	621	*Uranotaenia geometrica*	*Uranotaenia geometrica*	NC_044662.1	100.00%	100.00%	
Cx_tritaeniorhynchus_MG_S115	653	*Culex tritaeniorhynchus*	*Culex tritaeniorhynchus*	MK861440.1	100.00%	98.77%	
